# Supplementary material for: Identification and validation of a ferroptosis-related lncRNA signature to robustly predict the prognosis, immune microenvironment, and immunotherapy efficiency in patients with clear cell renal cell carcinoma
Source: PeerJ. 2022 Dec 19;10:e14506. doi: 10.7717/peerj.14506 (PMC9774008; doi:10.7717/peerj.14506)

	pvalue	Hazard ratio
SLC16A1−AS1	<0.001	3.781(2.310−6.189)
PCED1B−AS1	<0.001	1.184(1.096−1.278)
MMP25−AS1	<0.001	1.315(1.151−1.504)
LINC00894	<0.001	2.991(2.069−4.324)
DUXAP8	<0.001	4.211(2.554−6.943)
LINC01426	<0.001	1.084(1.047−1.123)
AL022322.1	<0.001	1.272(1.129−1.434)
ASMTL−AS1	<0.001	1.098(1.054−1.144)
AC005785.1	<0.001	1.589(1.299−1.945)
AC079322.1	<0.001	5.491(2.823−10.679)
PVT1	<0.001	1.287(1.184−1.399)
AL031714.1	<0.001	2.116(1.472−3.042)
USP30−AS1	<0.001	1.169(1.066−1.282)
MIR155HG	<0.001	1.105(1.061−1.152)
AC107021.2	0.001	1.128(1.048−1.213)
LINC01355	<0.001	1.874(1.561−2.250)
TRG−AS1	<0.001	1.681(1.277−2.211)
FAM13A−AS1	<0.001	1.397(1.158−1.685)
AL021707.6	0.001	1.115(1.045−1.190)
LINC00893	<0.001	1.686(1.281−2.220)
PELATON	<0.001	1.212(1.082−1.358)
AC007098.1	<0.001	2.126(1.479−3.056)
AL137186.2	<0.001	2.169(1.594−2.952)
LINC02609	<0.001	1.465(1.226−1.751)
HLA−DQB1−AS1	0.003	1.045(1.015−1.075)
ARHGAP27P1−BPTEP1−KPNA2P3	<0.001	1.355(1.159−1.584)
MYG1−AS1	<0.001	1.752(1.511−2.031)
PRKAR1B−AS1	<0.001	1.186(1.104−1.274)
MALAT1	<0.001	1.011(1.005−1.016)
LINC00342	<0.001	1.263(1.167−1.367)

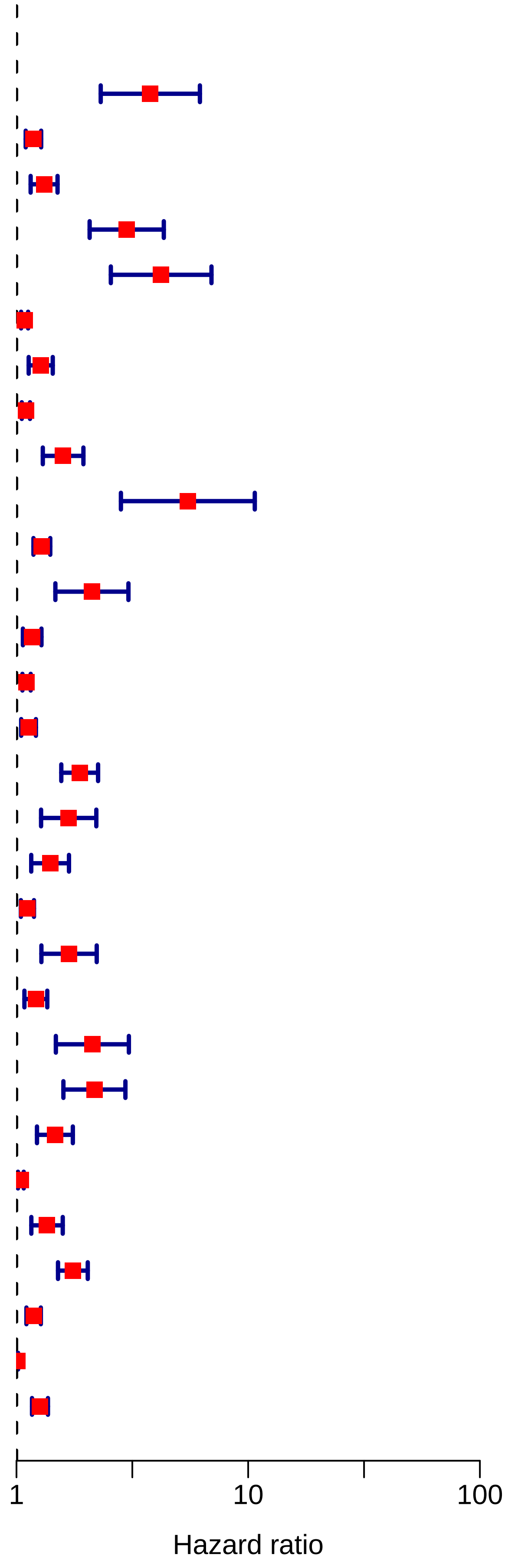

Supplement: Code S2 [file peerj-10-14506-s002.zip › 1. model construction/uni.foreast.pdf]
