# Supplementary material for: Identification and validation of a ferroptosis-related lncRNA signature to robustly predict the prognosis, immune microenvironment, and immunotherapy efficiency in patients with clear cell renal cell carcinoma
Source: PeerJ. 2022 Dec 19;10:e14506. doi: 10.7717/peerj.14506 (PMC9774008; doi:10.7717/peerj.14506)
Supplement: Code S2 [file peerj-10-14506-s002.zip › 6. risk vs cli/heatmap.pdf]

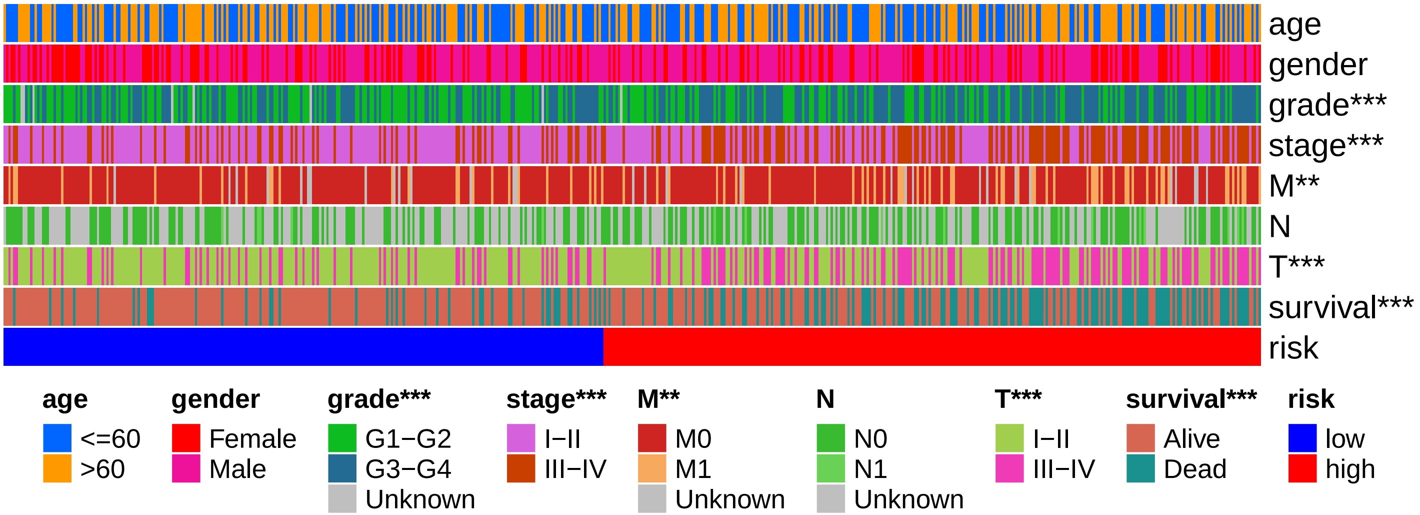
age
gender
grade***
stage***
M**
N
T***
survival***
risk
age
<=60
>60
gender
Female
Male
grade***
G1−G2
G3−G4
Unknown
stage***
I−II
III−IV
M**
M0
M1
Unknown
N
N0
N1
Unknown
T***
I−II
III−IV
survival***
Alive
Dead
risk
low
high
