# Supplementary figures and images for: Identification and validation of a ferroptosis-related lncRNA signature to robustly predict the prognosis, immune microenvironment, and immunotherapy efficiency in patients with clear cell renal cell carcinoma
Source: PeerJ. 2022 Dec 19;10:e14506. doi: 10.7717/peerj.14506 (PMC9774008; doi:10.7717/peerj.14506)

Partial Likelihood Deviance

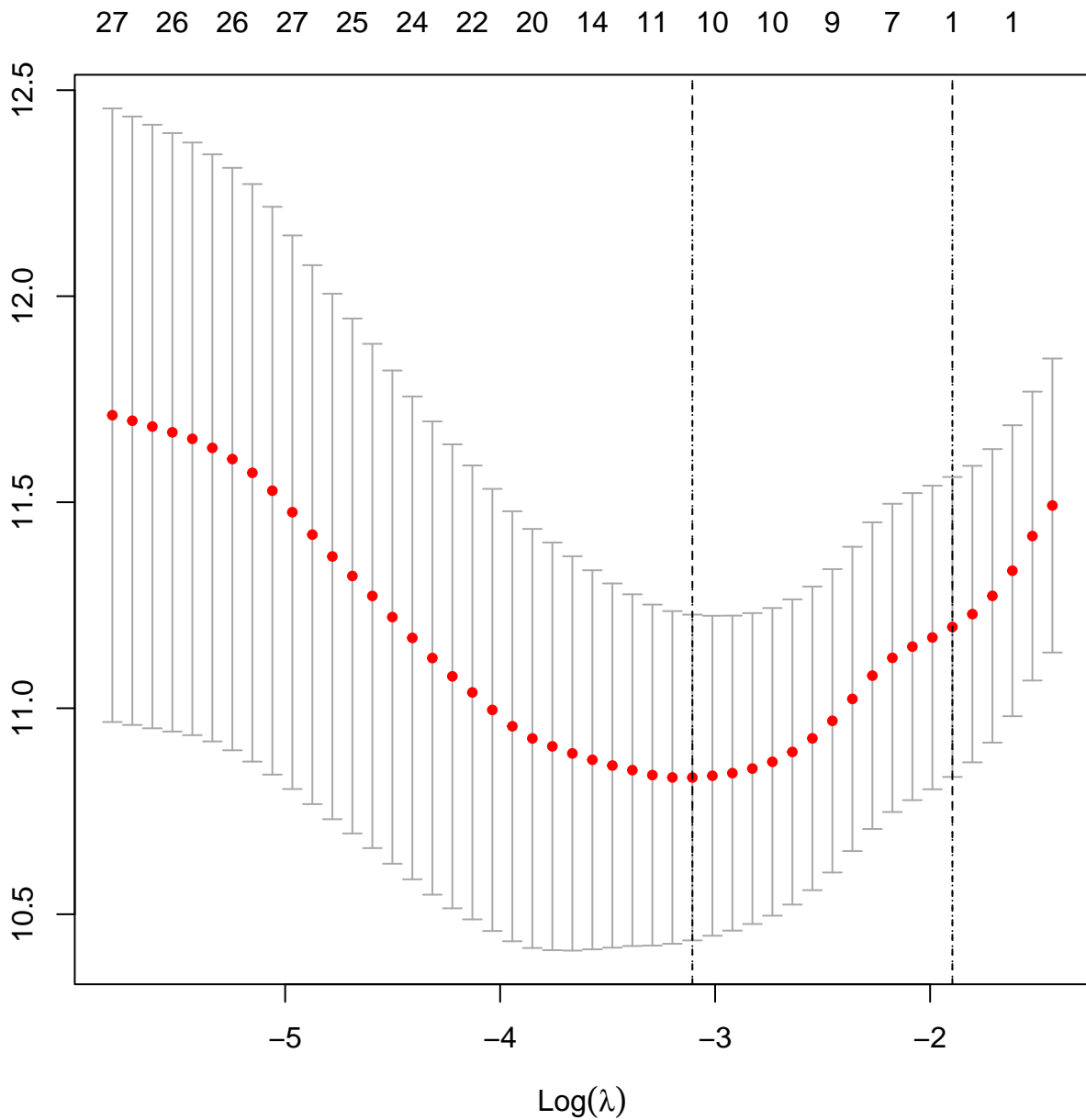

Supplement: Code S2 [file peerj-10-14506-s002.zip › 1. model construction/lasso.cvfit.pdf]

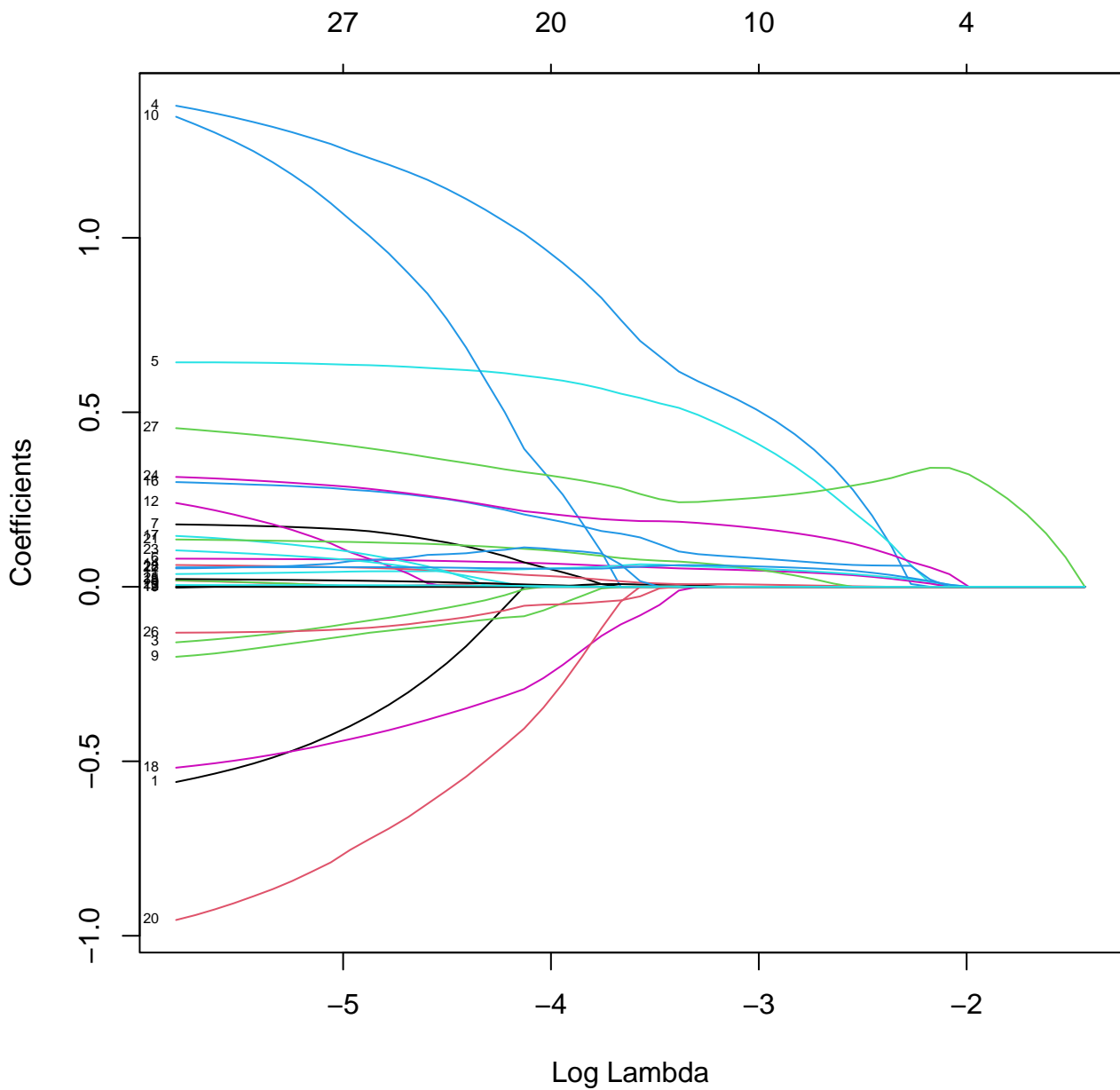

Supplement: Code S2 [file peerj-10-14506-s002.zip › 1. model construction/lasso.lambda.pdf]

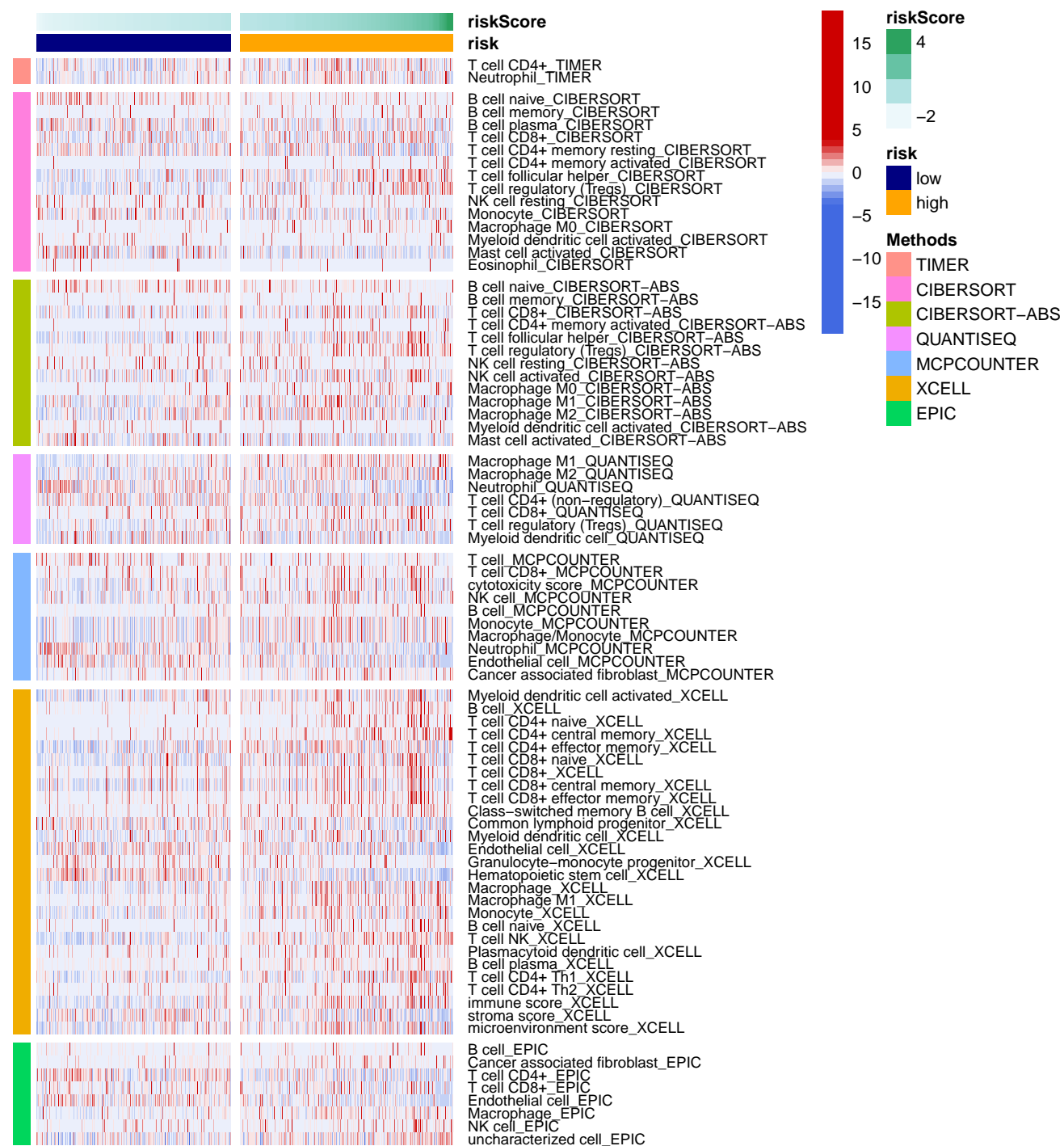

Methods

Supplement: Code S2 [file peerj-10-14506-s002.zip › 10. immuneHeatmap/immHeatmap.pdf]

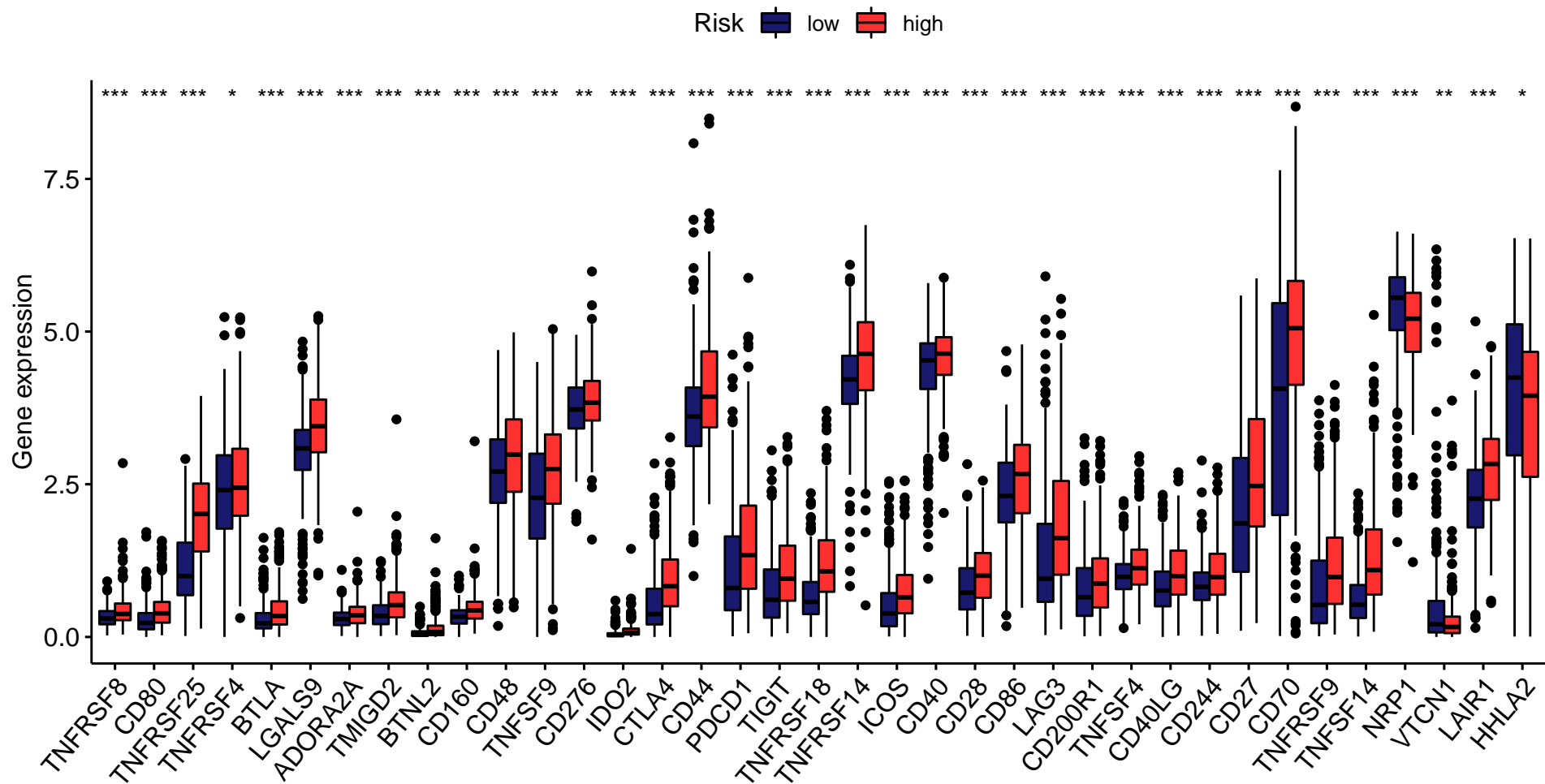

Supplement: Code S2 [file peerj-10-14506-s002.zip › 11. risk vs immune checkpoints/checkpoint.pdf]

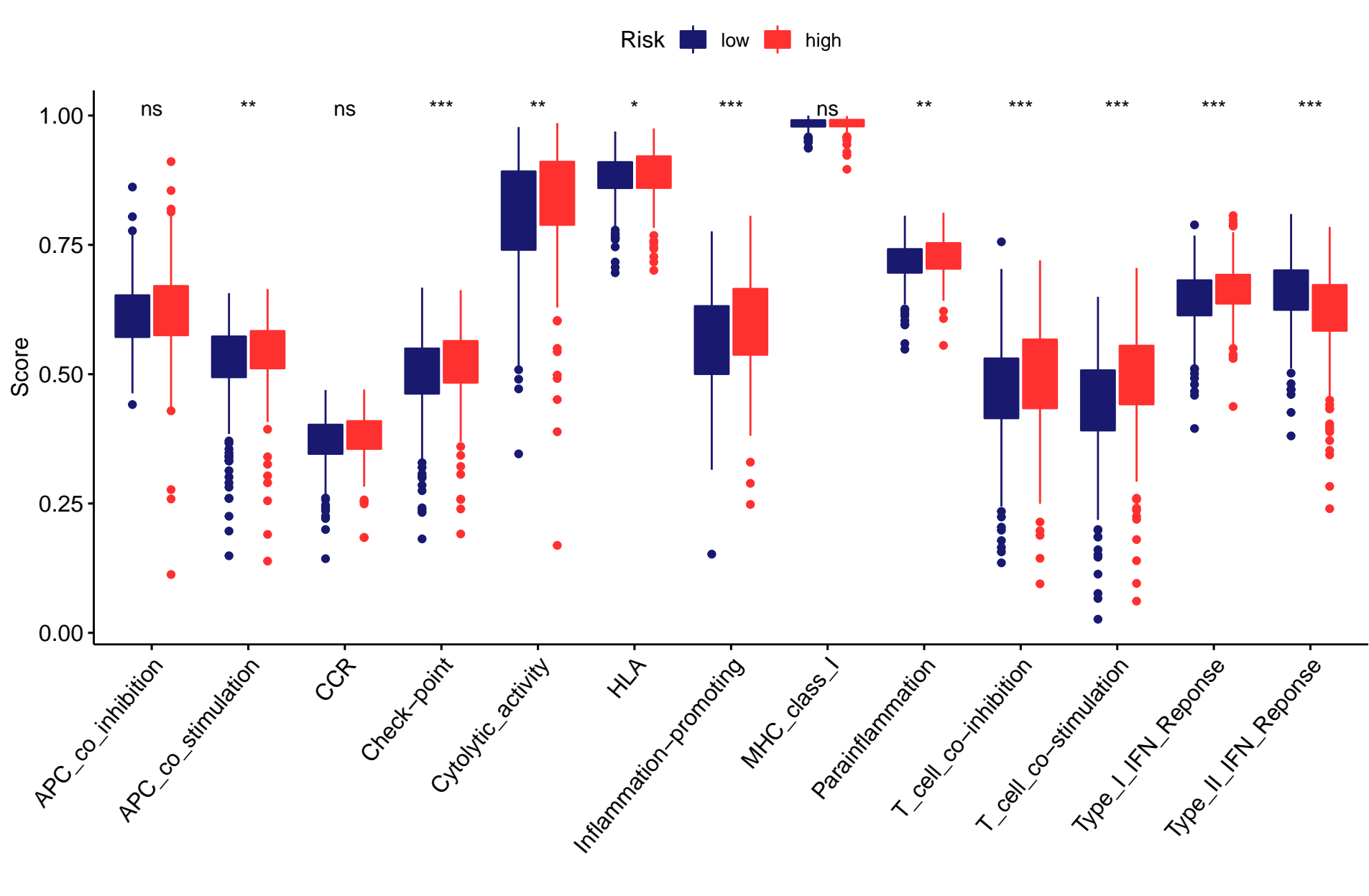

Supplement: Code S2 [file peerj-10-14506-s002.zip › 11. risk vs immune checkpoints/ssGSEA.pdf]

Risk 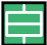 low 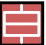 high

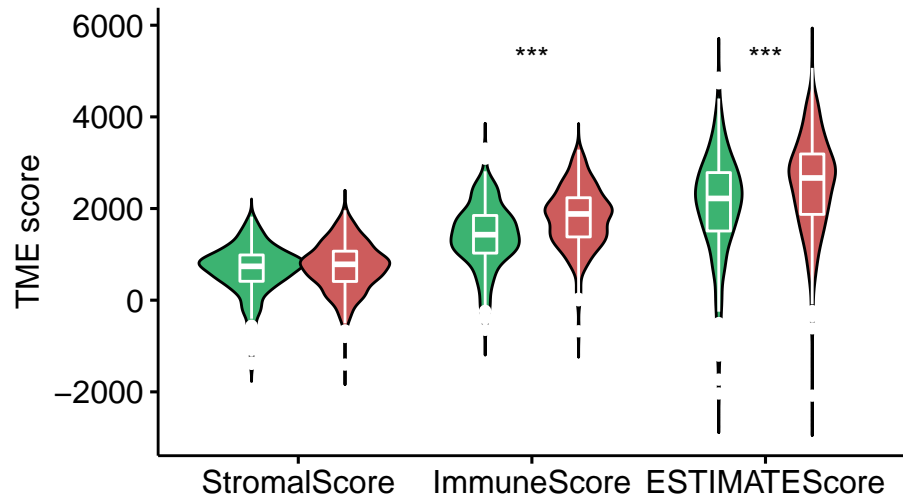

Supplement: Code S2 [file peerj-10-14506-s002.zip › 12. estimate/estimate.pdf]

risk High-risk Low-risk

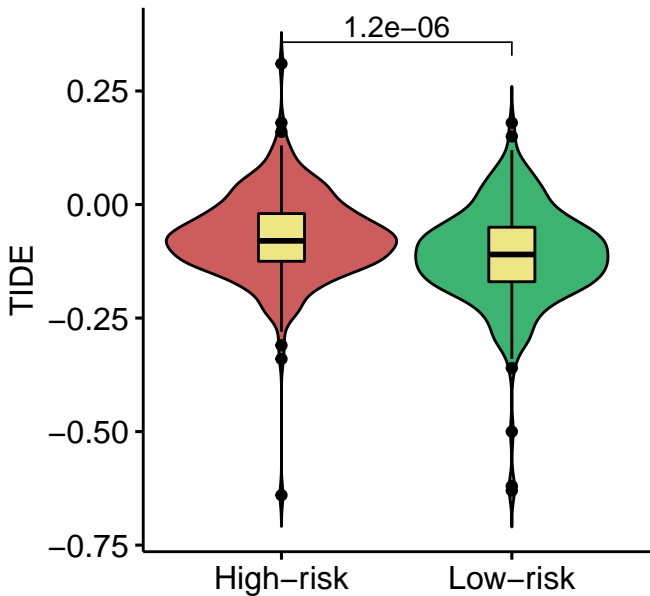

Supplement: Code S2 [file peerj-10-14506-s002.zip › 13. TIDE/TIDE.pdf]

Response

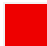

Non-responder

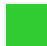

Responder

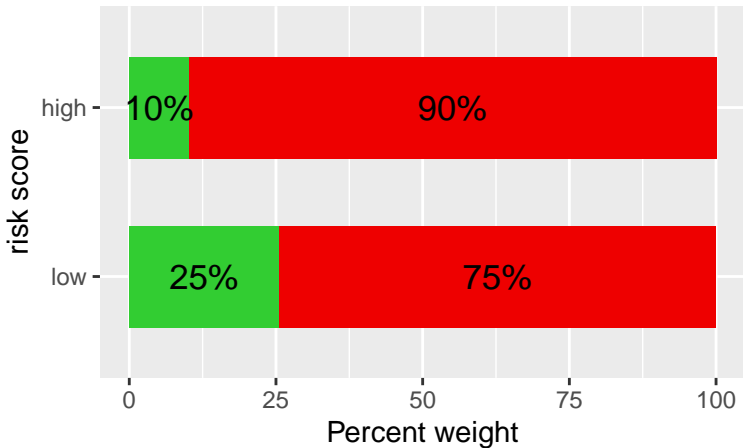

Supplement: Code S2 [file peerj-10-14506-s002.zip › 14. ImmuCellAI/ImmuCellAI.PercentWeight.pdf]

Response 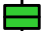 Responder 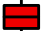 Non-responder

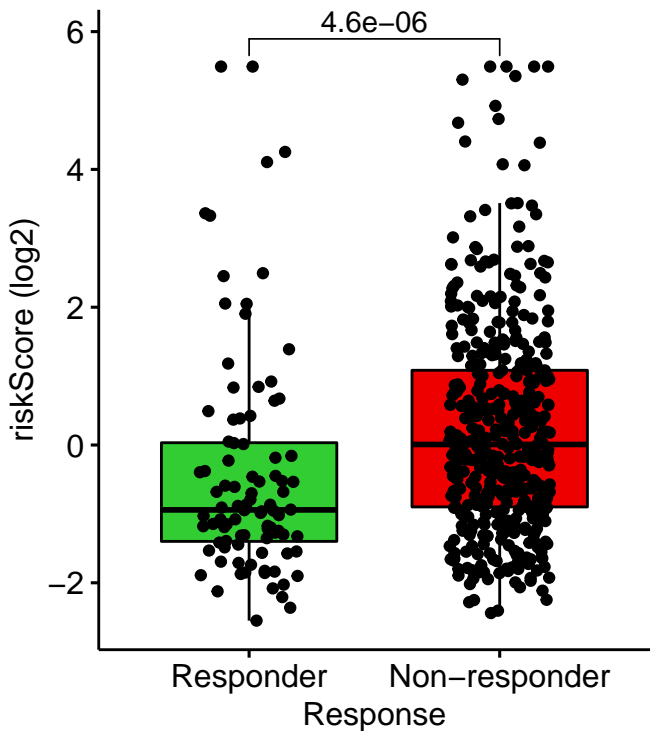

Supplement: Code S2 [file peerj-10-14506-s002.zip › 14. ImmuCellAI/ImmuCellAI.scatter.pdf]

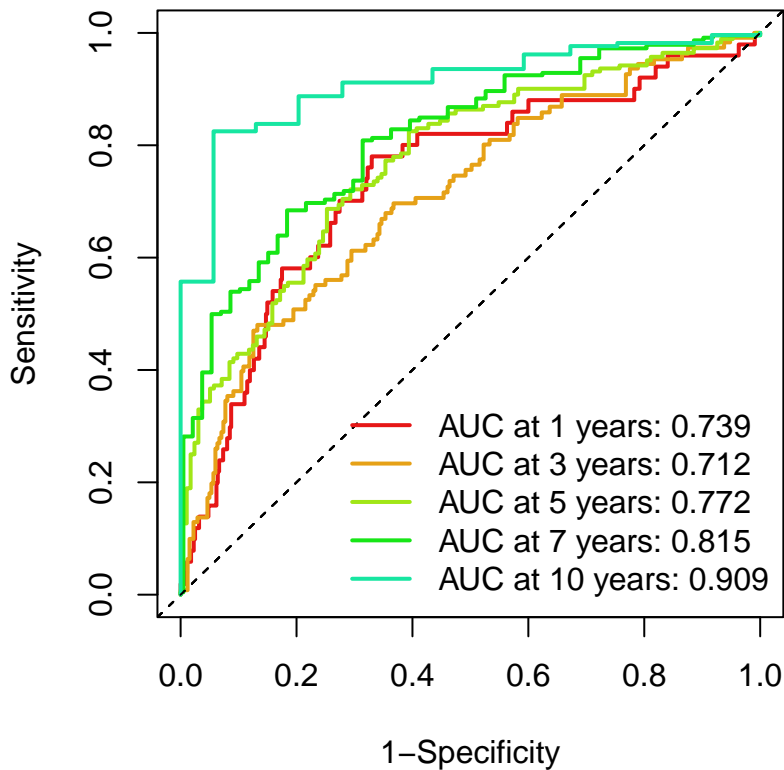

Supplement: Code S2 [file peerj-10-14506-s002.zip › 2. ROC et km/all.ROC.pdf]

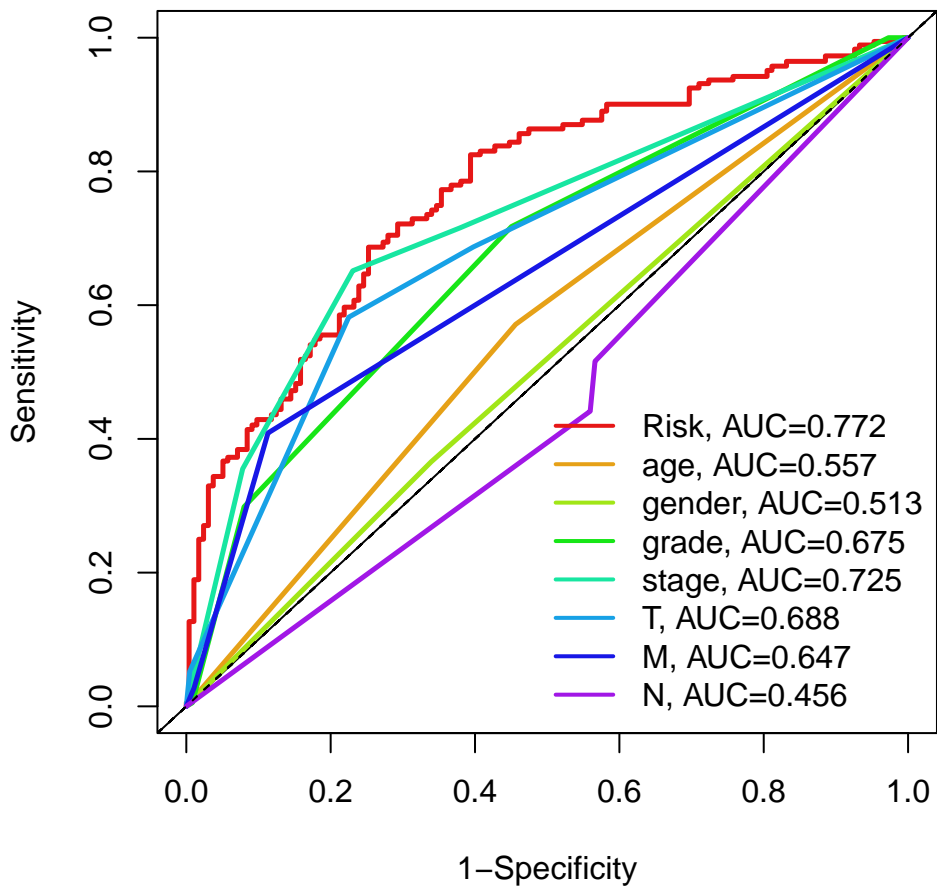

Supplement: Code S2 [file peerj-10-14506-s002.zip › 2. ROC et km/all.cliROC.pdf]

Overall survival

Risk + High risk + Low risk

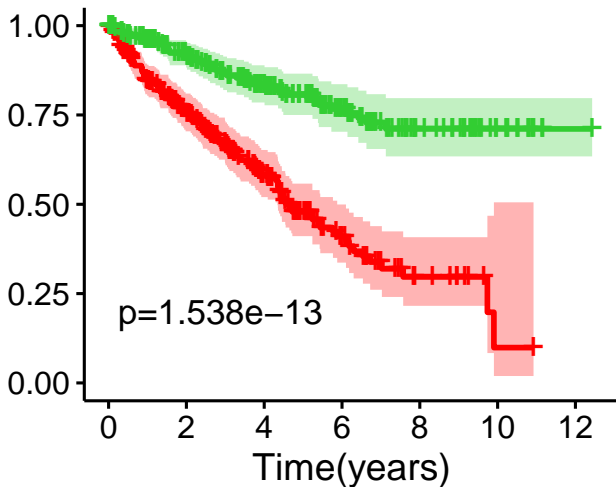

Risk

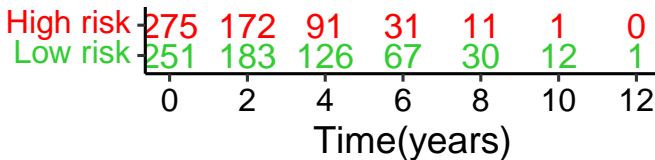

Supplement: Code S2 [file peerj-10-14506-s002.zip › 2. ROC et km/surv.all.pdf]

Overall survival

Risk 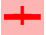 High risk 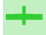 Low risk

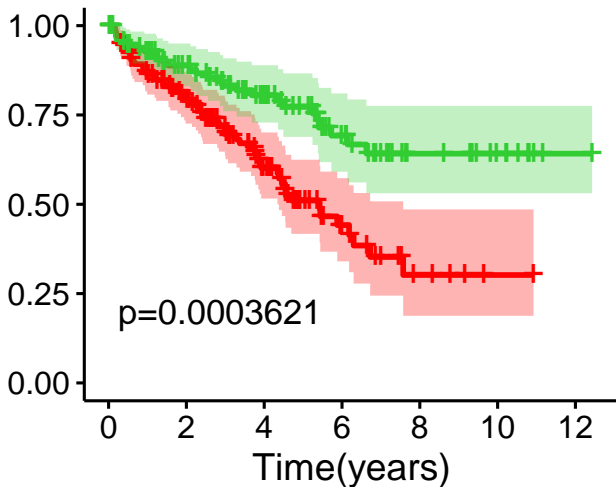

Risk

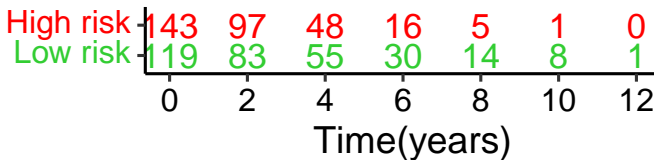

Supplement: Code S2 [file peerj-10-14506-s002.zip › 2. ROC et km/surv.test.pdf]

Overall survival

Risk + High risk + Low risk

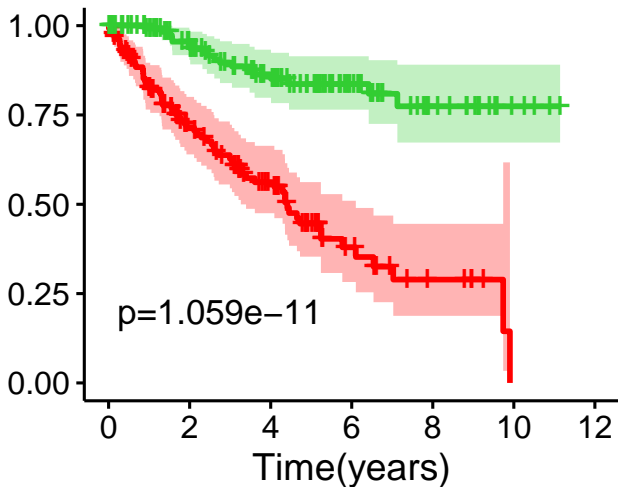

Risk

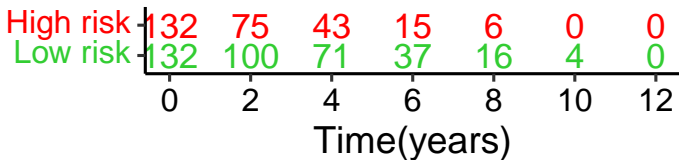

Supplement: Code S2 [file peerj-10-14506-s002.zip › 2. ROC et km/surv.train.pdf]

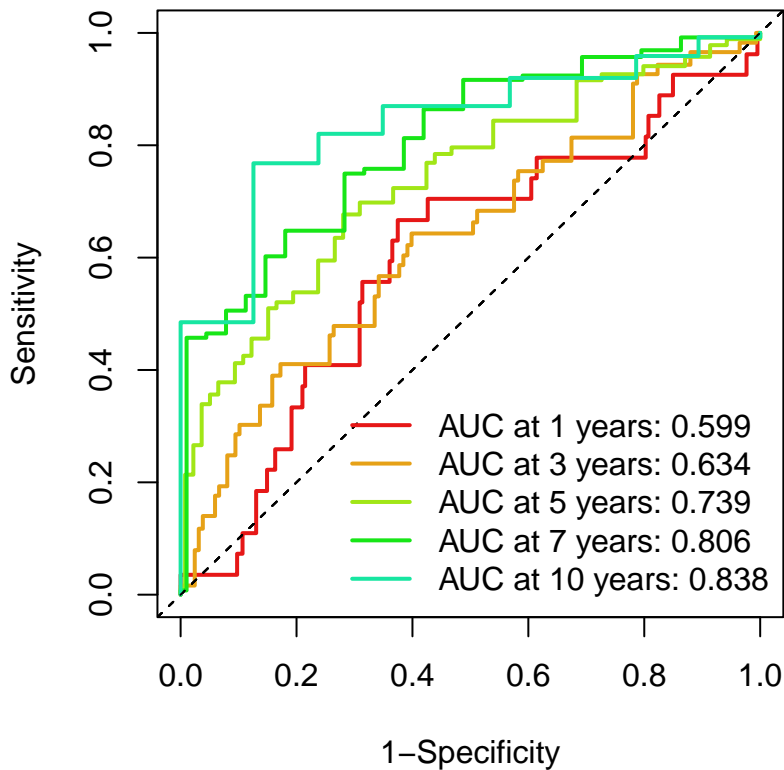

Supplement: Code S2 [file peerj-10-14506-s002.zip › 2. ROC et km/test.ROC.pdf]

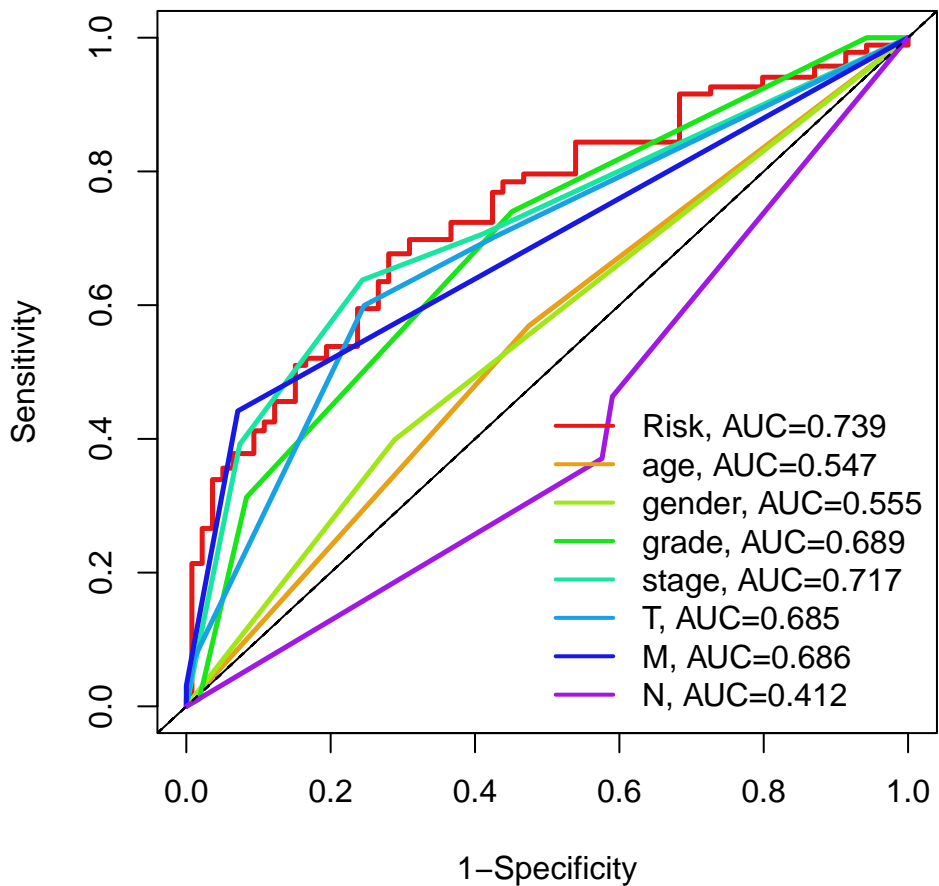

Supplement: Code S2 [file peerj-10-14506-s002.zip › 2. ROC et km/test.cliROC.pdf]

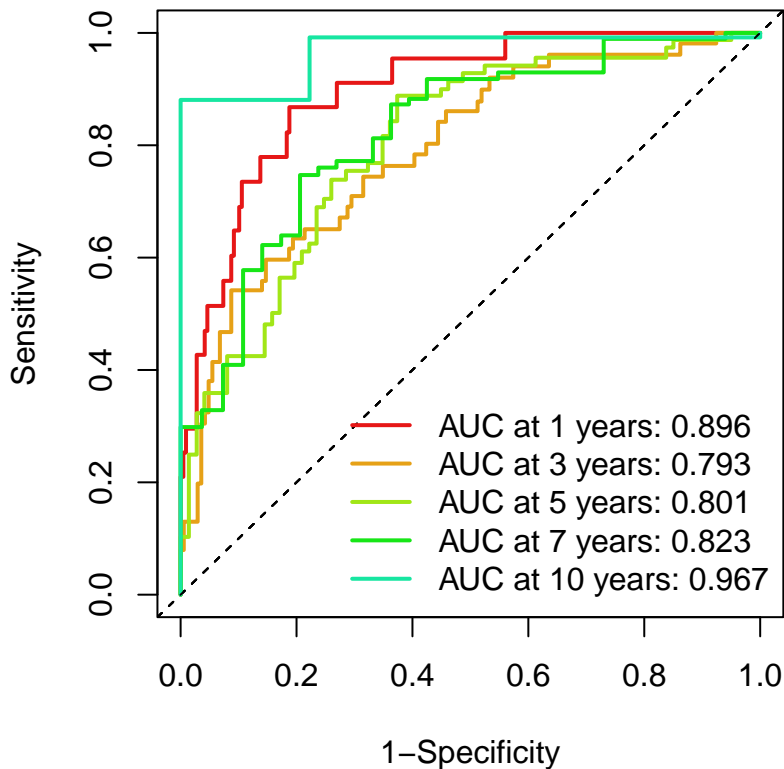

Supplement: Code S2 [file peerj-10-14506-s002.zip › 2. ROC et km/train.ROC.pdf]

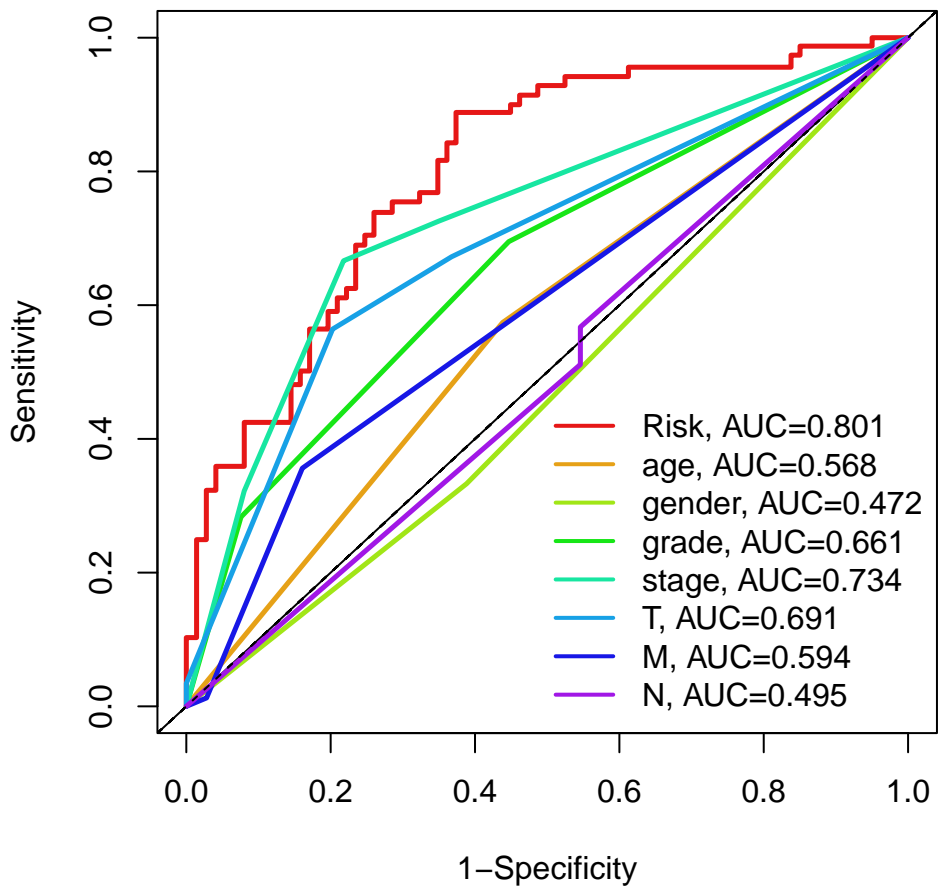

Supplement: Code S2 [file peerj-10-14506-s002.zip › 2. ROC et km/train.cliROC.pdf]

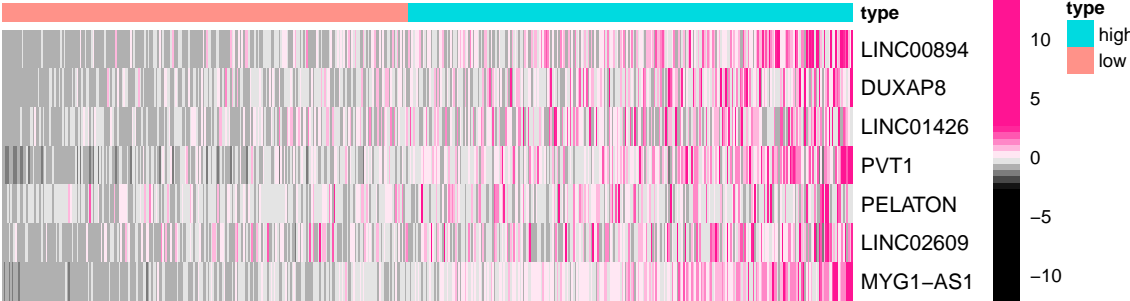

Supplement: Code S2 [file peerj-10-14506-s002.zip › 3. riskplot/all.heatmap.pdf]

Risk score

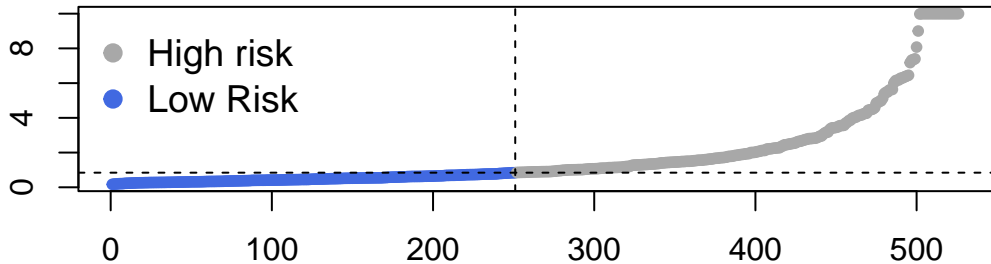

Patients (increasing risk socre)

Supplement: Code S2 [file peerj-10-14506-s002.zip › 3. riskplot/all.riskScore.pdf]

Survival time (years)

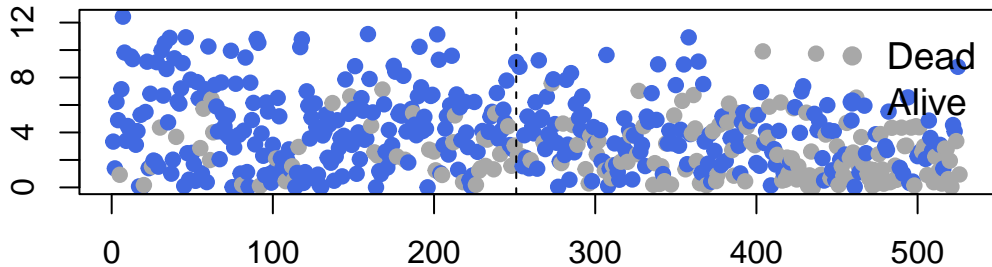

Patients (increasing risk socre)

Supplement: Code S2 [file peerj-10-14506-s002.zip › 3. riskplot/all.survStat.pdf]

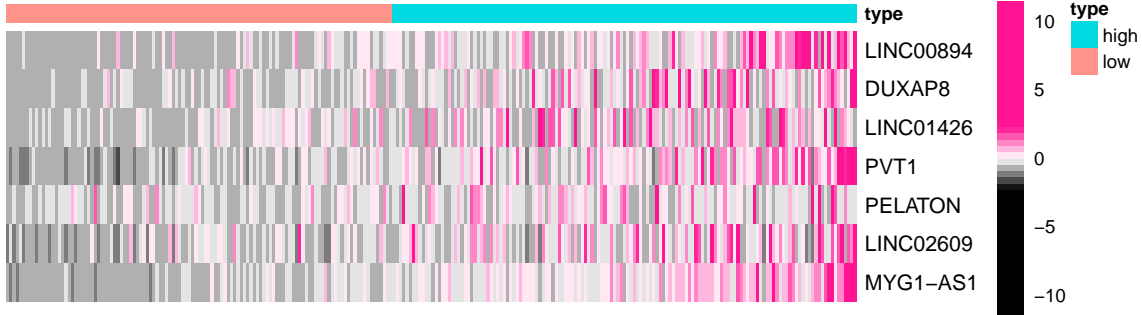

Supplement: Code S2 [file peerj-10-14506-s002.zip › 3. riskplot/test.heatmap.pdf]

Risk score

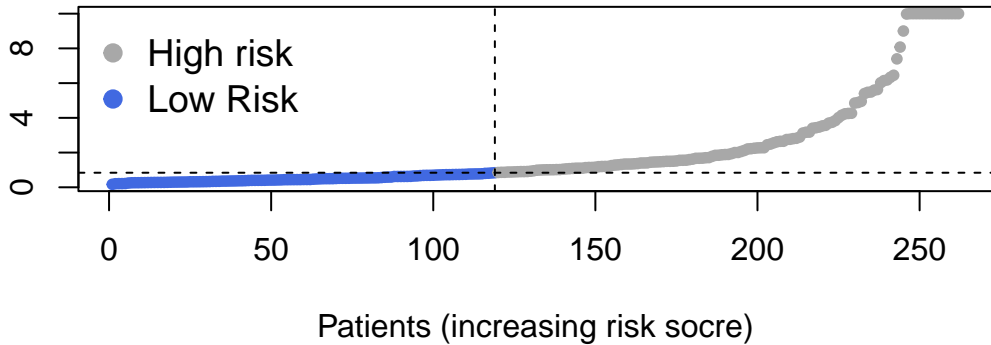

Supplement: Code S2 [file peerj-10-14506-s002.zip › 3. riskplot/test.riskScore.pdf]

Survival time (years)

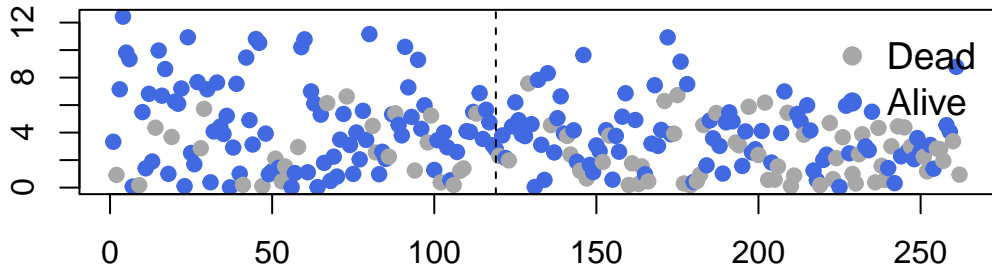

Patients (increasing risk score)

Supplement: Code S2 [file peerj-10-14506-s002.zip › 3. riskplot/test.survStat.pdf]

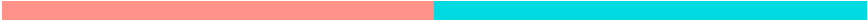

type

type

high

low

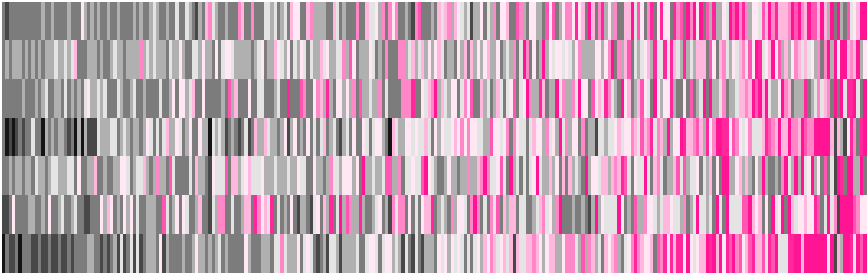

LINC00894

DUXAP8

LINC01426

PVT1

PELATON

LINC02609

MYG1-AS1

5

0

-5

Supplement: Code S2 [file peerj-10-14506-s002.zip › 3. riskplot/train.heatmap.pdf]

Risk score

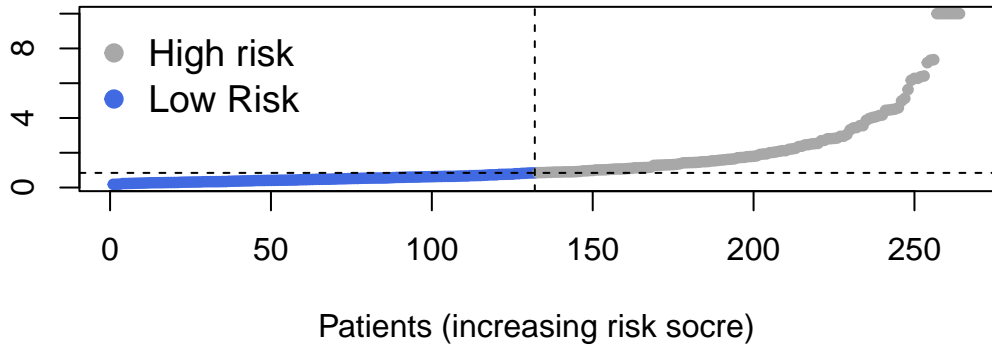

Supplement: Code S2 [file peerj-10-14506-s002.zip › 3. riskplot/train.riskScore.pdf]

Survival time (years)

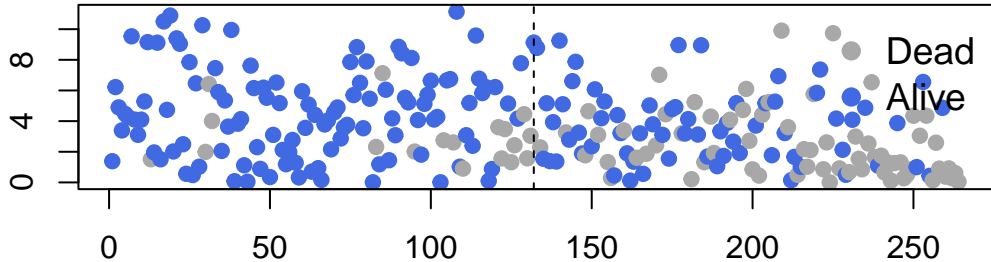

Patients (increasing risk socre)

Supplement: Code S2 [file peerj-10-14506-s002.zip › 3. riskplot/train.survStat.pdf]

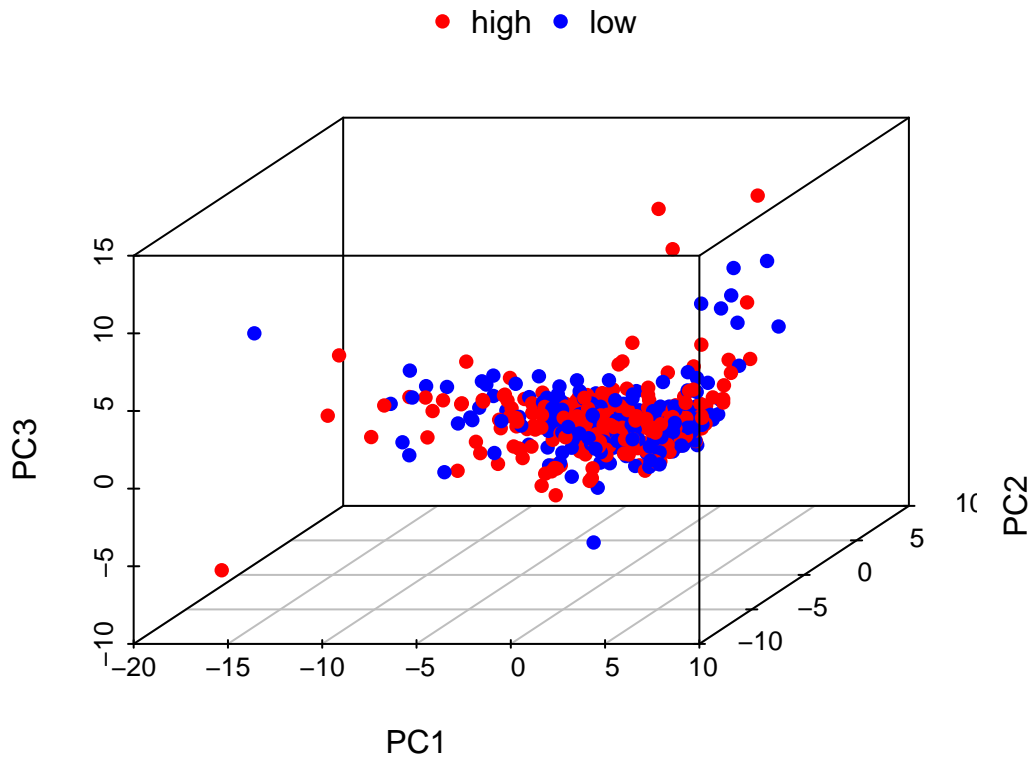

Supplement: Code S2 [file peerj-10-14506-s002.zip › 4. PCA/PCA.LncRNA.3d.pdf]

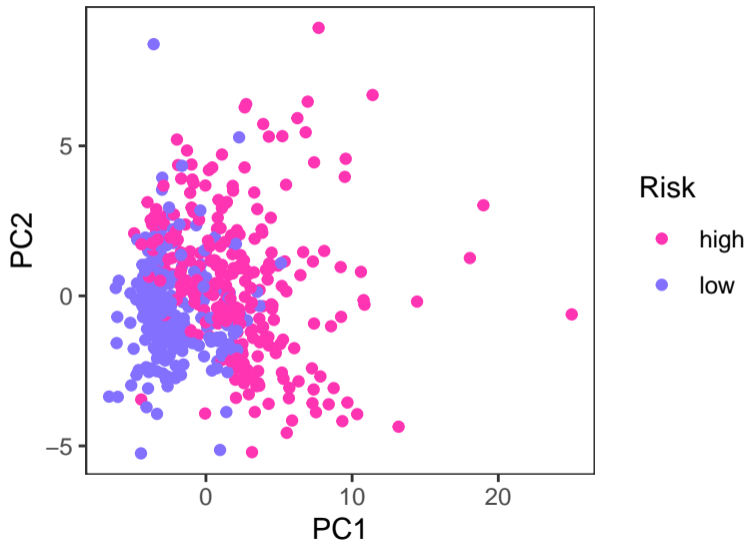

Supplement: Code S2 [file peerj-10-14506-s002.zip › 4. PCA/PCA.LncRNA.pdf]

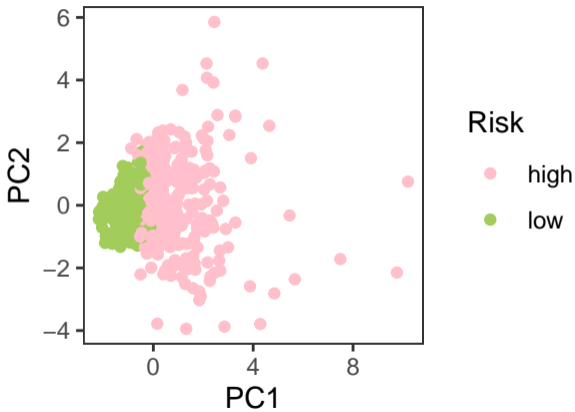

Supplement: Code S2 [file peerj-10-14506-s002.zip › 4. PCA/PCA.all.risk.pdf]

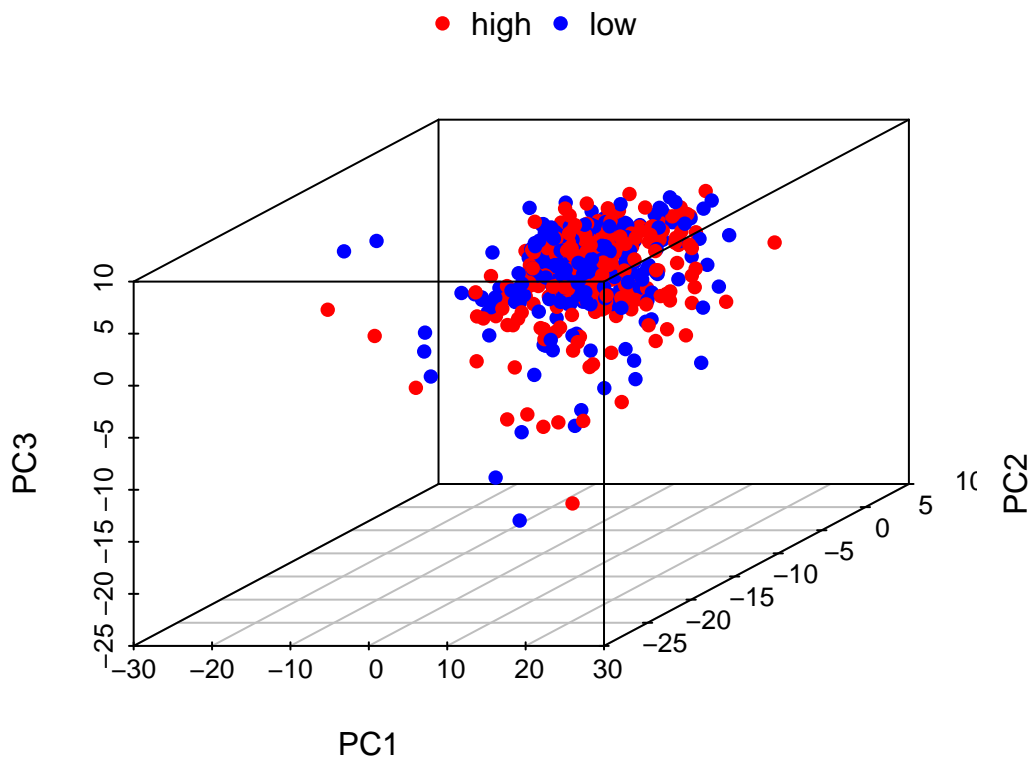

Supplement: Code S2 [file peerj-10-14506-s002.zip › 4. PCA/PCA.ferGene.3d.pdf]

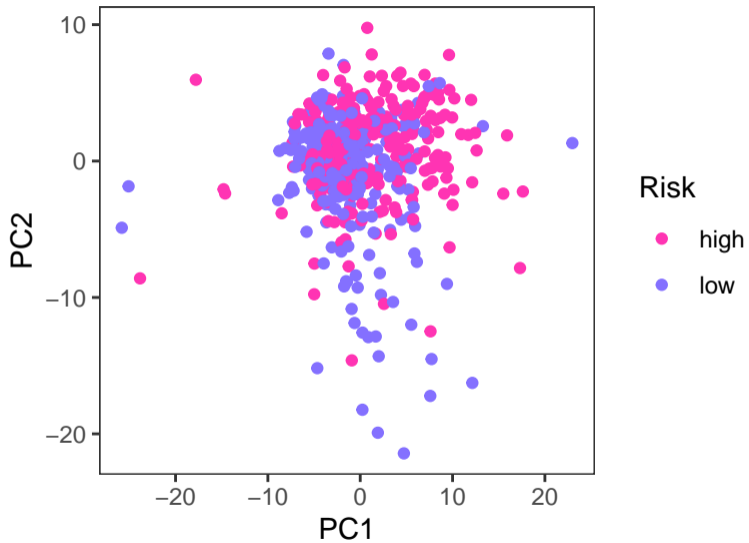

Supplement: Code S2 [file peerj-10-14506-s002.zip › 4. PCA/PCA.ferGene.pdf]

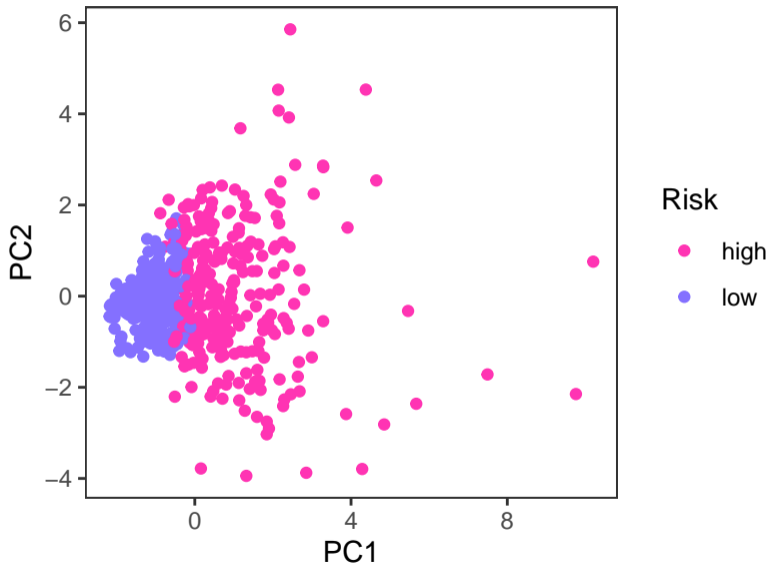

Supplement: Code S2 [file peerj-10-14506-s002.zip › 4. PCA/PCA.risk.pdf]

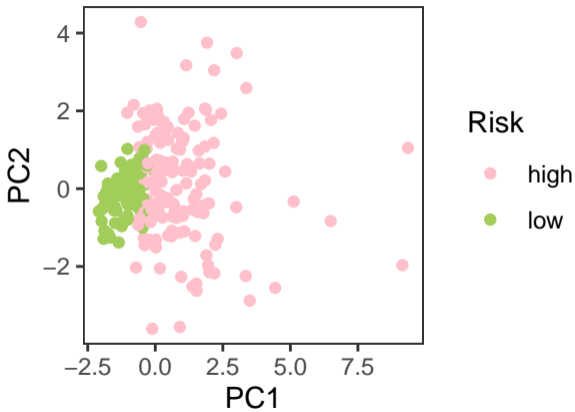

Supplement: Code S2 [file peerj-10-14506-s002.zip › 4. PCA/PCA.test.risk.pdf]

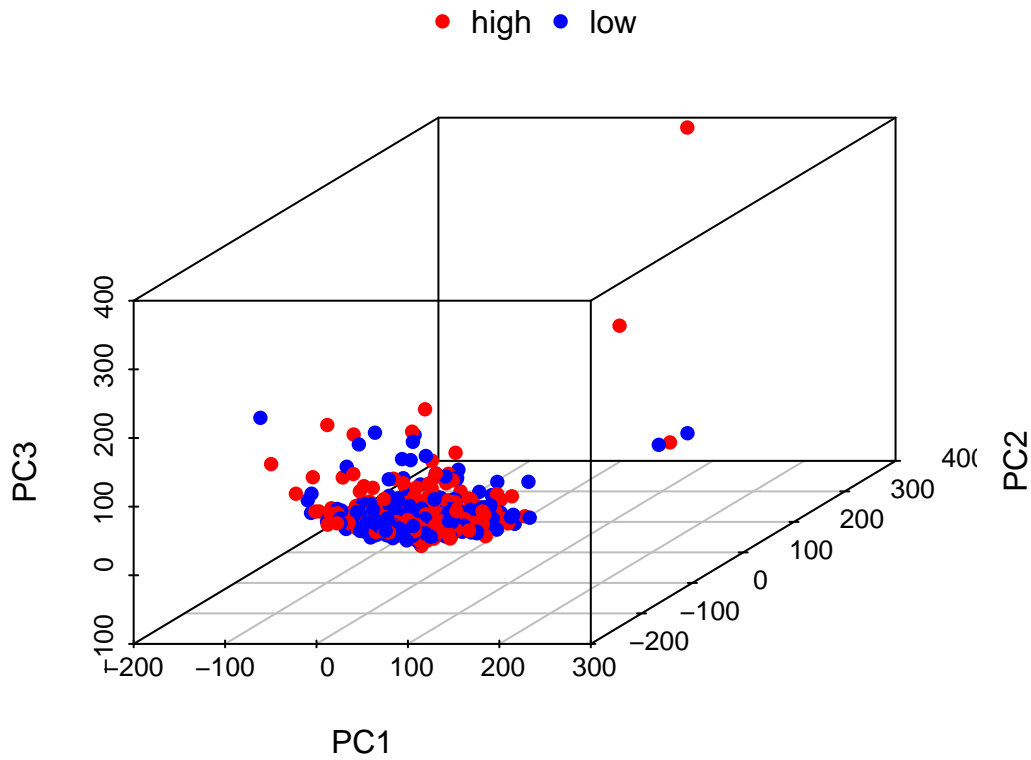

Supplement: Code S2 [file peerj-10-14506-s002.zip › 4. PCA/PCA.total.3d.pdf]

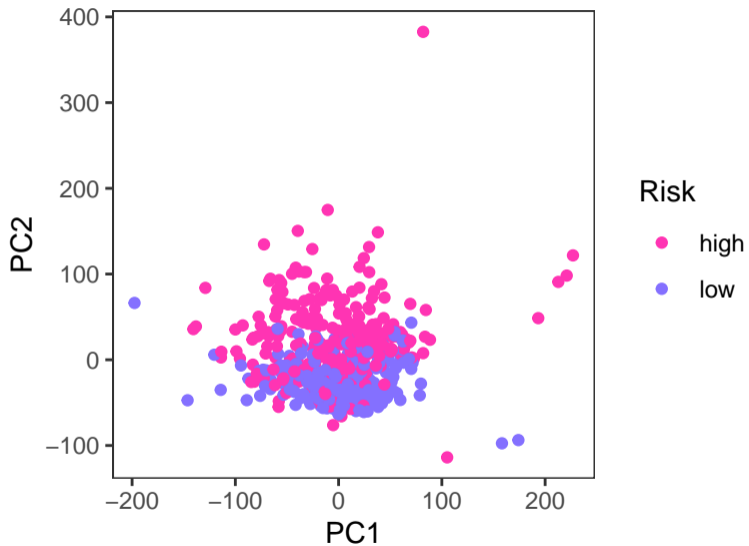

Supplement: Code S2 [file peerj-10-14506-s002.zip › 4. PCA/PCA.total.pdf]

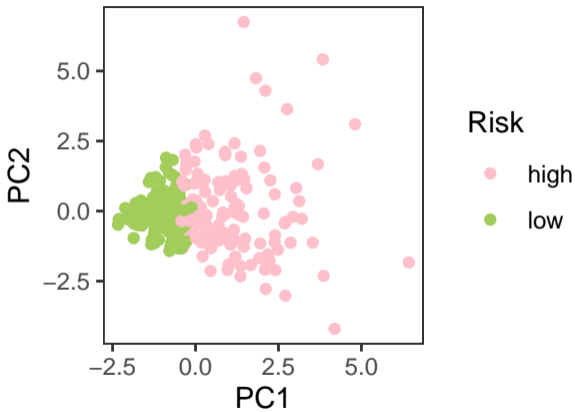

Supplement: Code S2 [file peerj-10-14506-s002.zip › 4. PCA/PCA.train.risk.pdf]

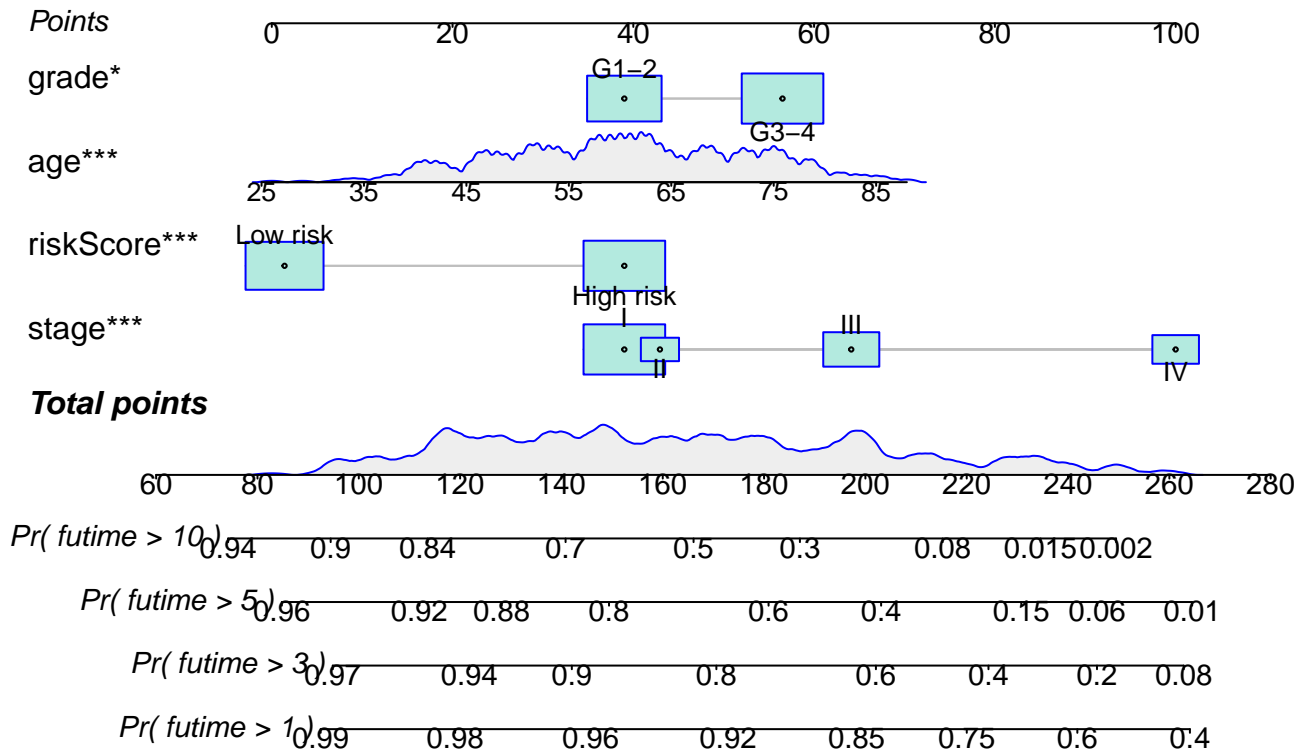

Supplement: Code S2 [file peerj-10-14506-s002.zip › 5. Nomogram/Nomogram.highlowRisk.pdf]

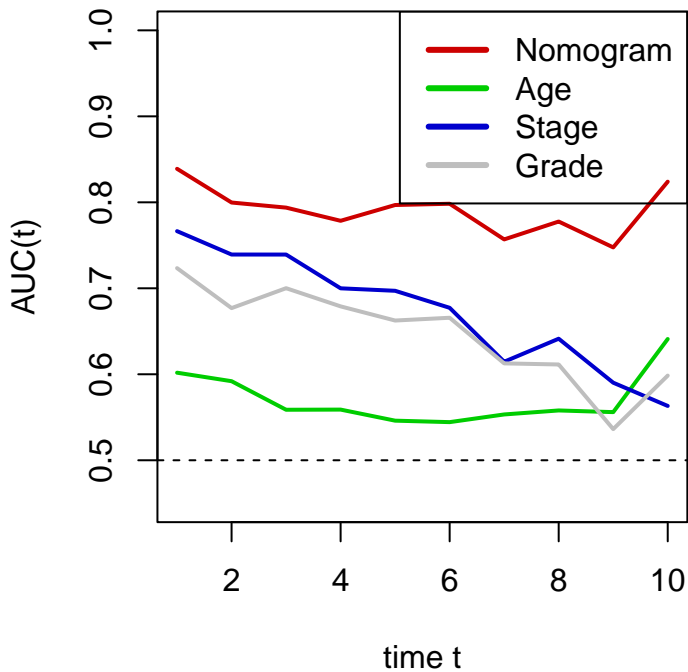

Supplement: Code S2 [file peerj-10-14506-s002.zip › 5. Nomogram/TimeDependentAUC.age12.stage12.pdf]

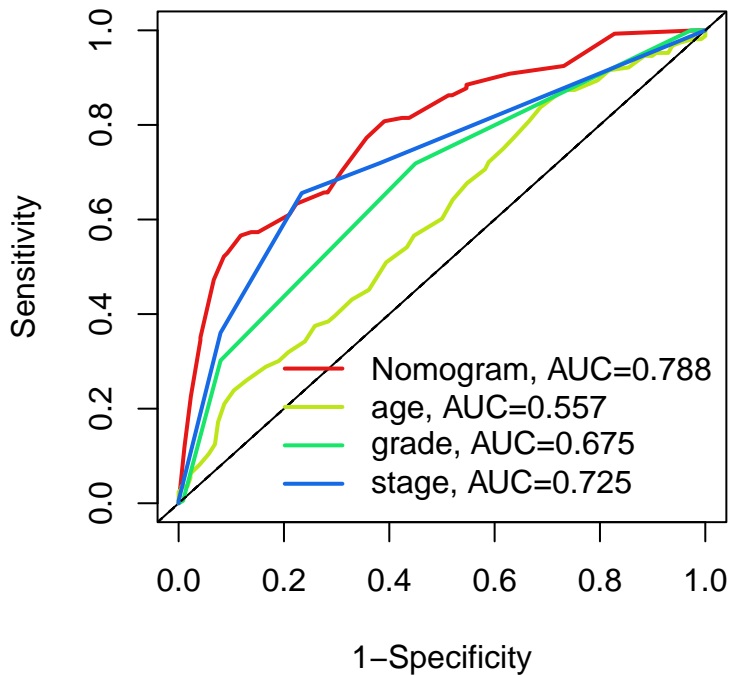

Supplement: Code S2 [file peerj-10-14506-s002.zip › 5. Nomogram/cliROC_5.age12.pdf]

Standardized Net Benefit

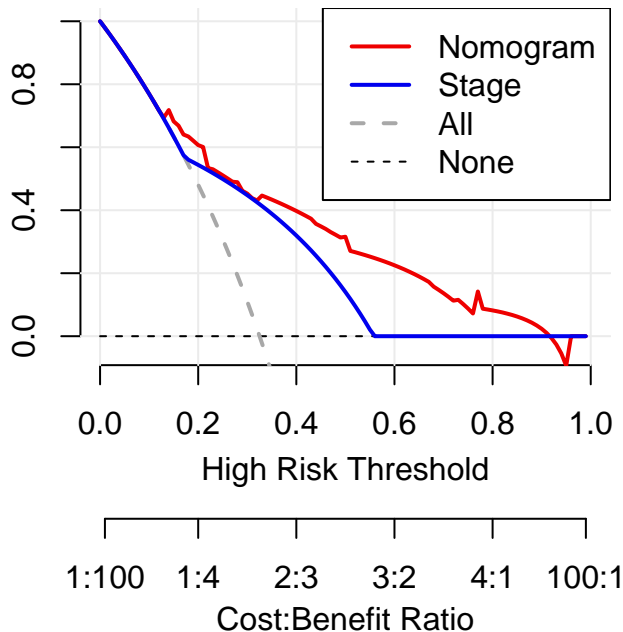

Supplement: Code S2 [file peerj-10-14506-s002.zip › 5. Nomogram/risk║≤.DCA.age12.pdf]

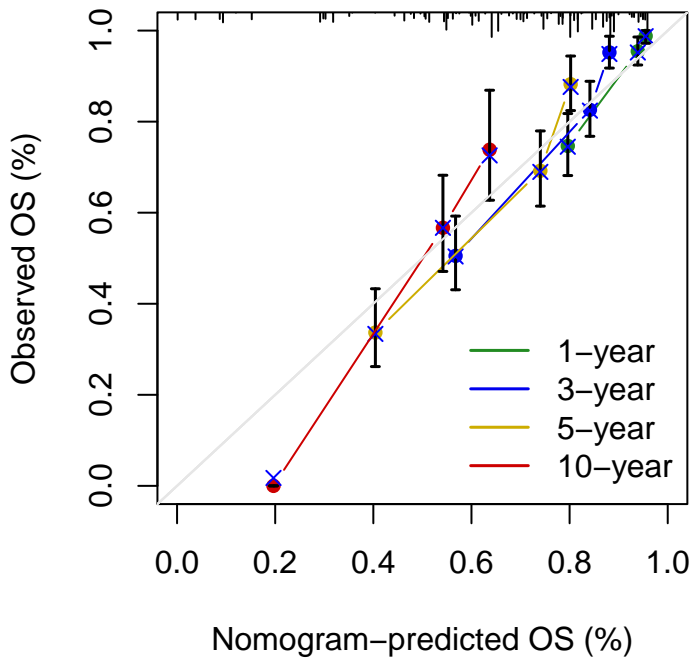

Supplement: Code S2 [file peerj-10-14506-s002.zip › 5. Nomogram/risk║≤.calibration.age12.pdf]

M M0 M1 Unknown

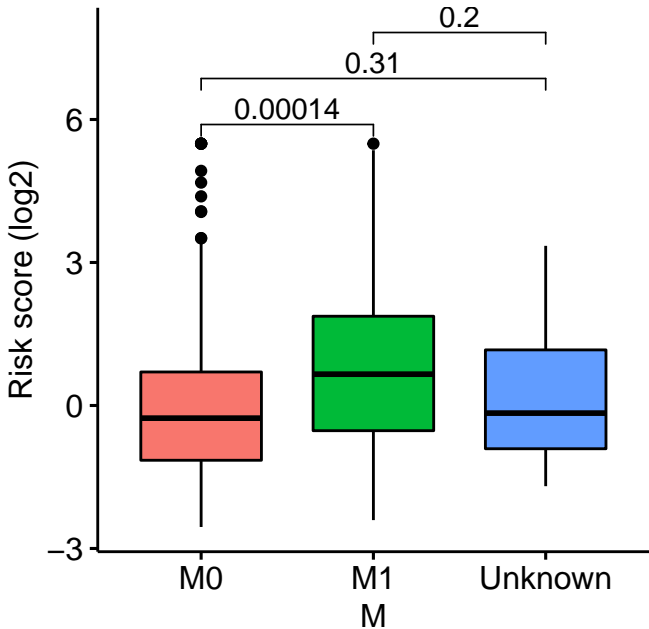

Supplement: Code S2 [file peerj-10-14506-s002.zip › 6. risk vs cli/clinicalCor_M.pdf]

N N0 N1 Unknown

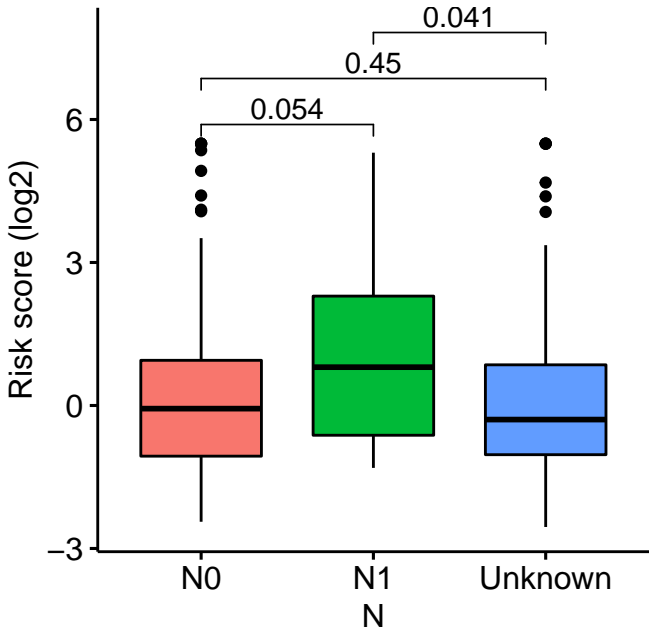

Supplement: Code S2 [file peerj-10-14506-s002.zip › 6. risk vs cli/clinicalCor_N.pdf]

T I-II III-IV

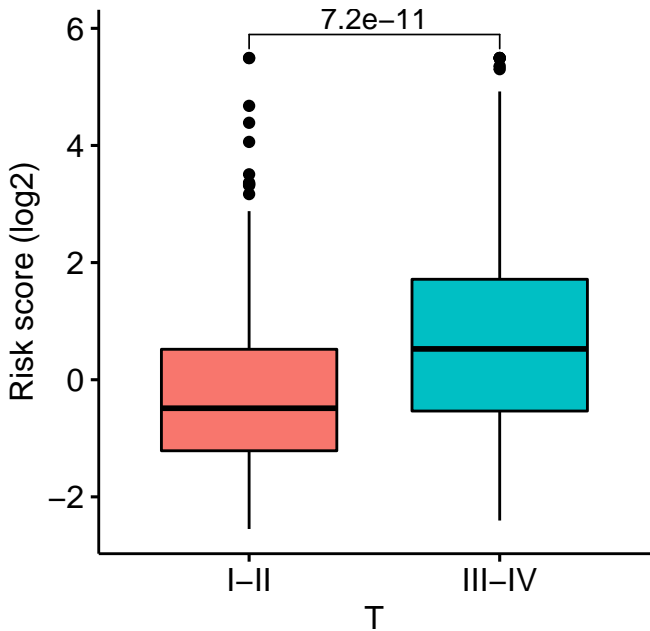

Supplement: Code S2 [file peerj-10-14506-s002.zip › 6. risk vs cli/clinicalCor_T.pdf]

age    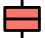  $\leq 60$     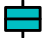  $> 60$

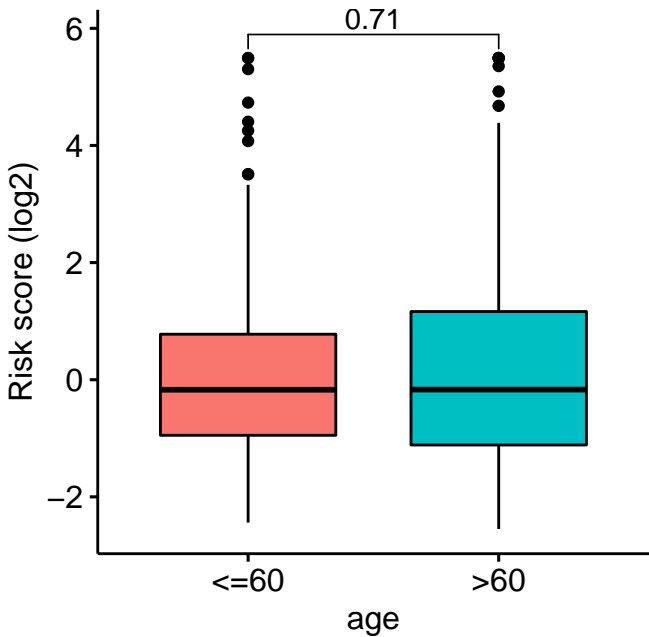

Supplement: Code S2 [file peerj-10-14506-s002.zip › 6. risk vs cli/clinicalCor_age.pdf]

gender Female Male

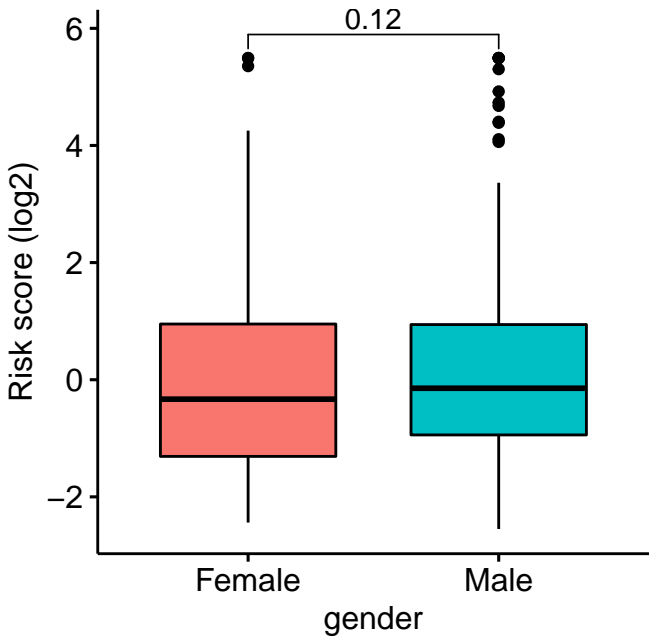

Supplement: Code S2 [file peerj-10-14506-s002.zip › 6. risk vs cli/clinicalCor_gender.pdf]

grade 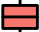 G1-G2 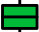 G3-G4 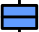 Unknown

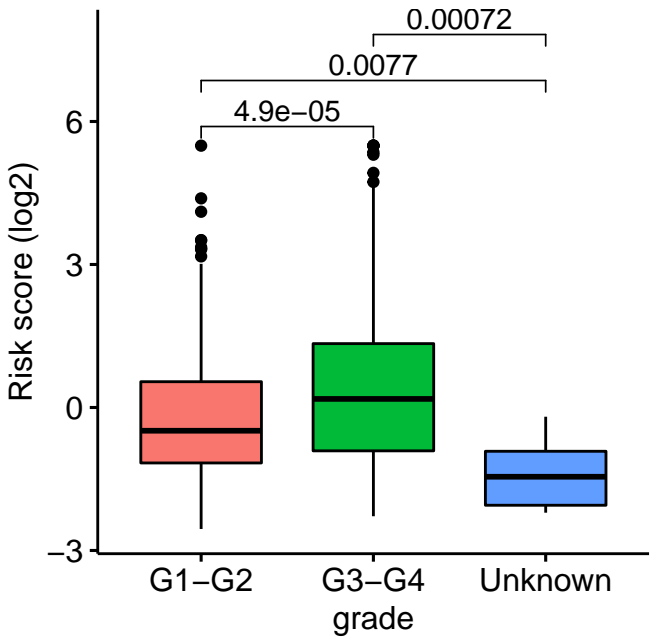

Supplement: Code S2 [file peerj-10-14506-s002.zip › 6. risk vs cli/clinicalCor_grade.pdf]

risk high low

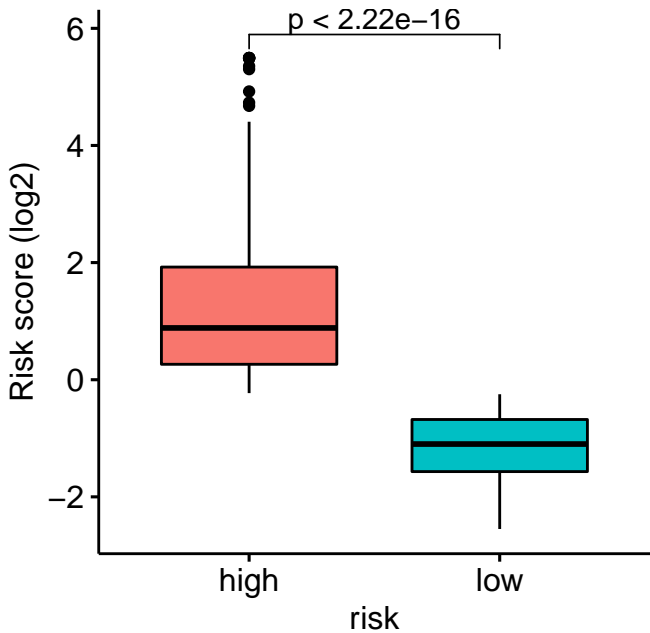

Supplement: Code S2 [file peerj-10-14506-s002.zip › 6. risk vs cli/clinicalCor_risk.pdf]

stage I-II III-IV

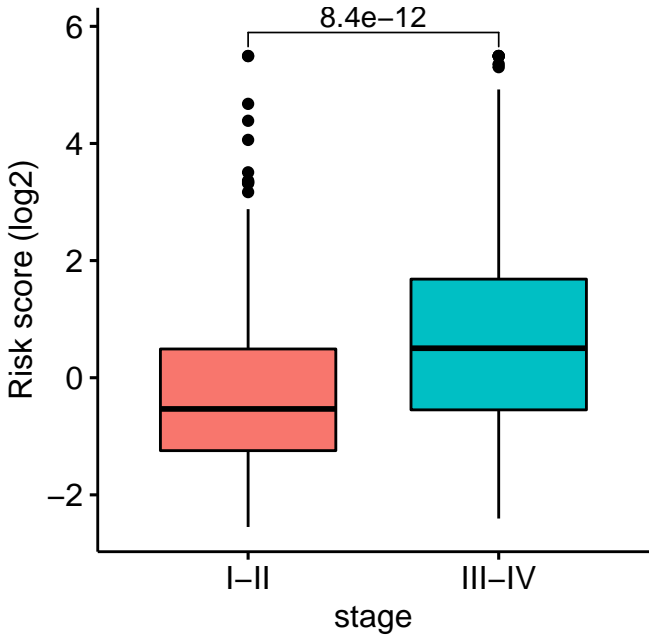

Supplement: Code S2 [file peerj-10-14506-s002.zip › 6. risk vs cli/clinicalCor_stage.pdf]

survival Alive Dead

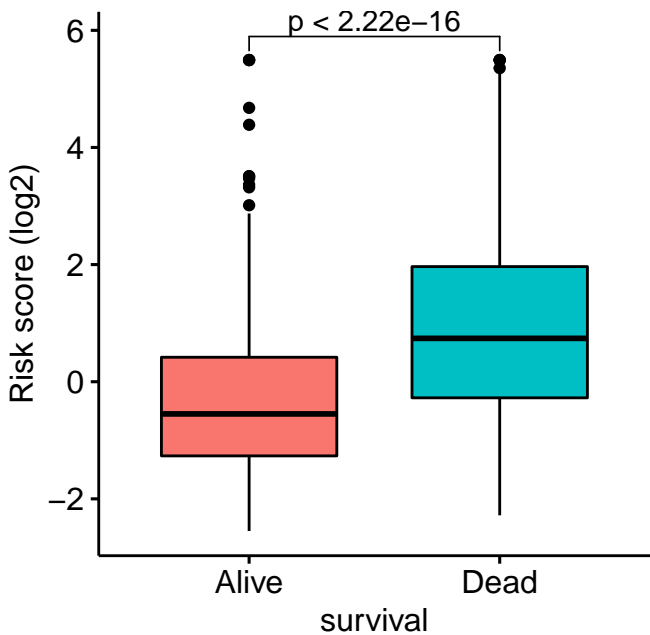

Supplement: Code S2 [file peerj-10-14506-s002.zip › 6. risk vs cli/clinicalCor_survival.pdf]

# Patients with M0

Risk 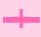 high 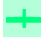 low

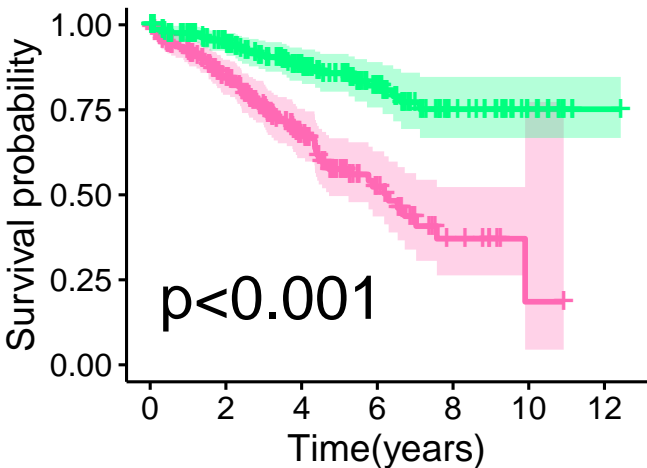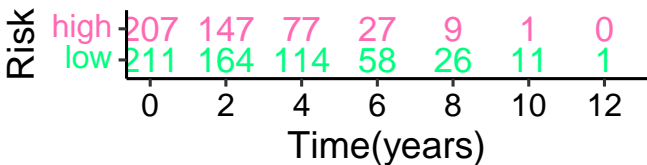

Supplement: Code S2 [file peerj-10-14506-s002.zip › 7. risk vs km/sur.M_M0.pdf]

# Patients with M1

Risk 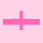 high 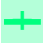 low

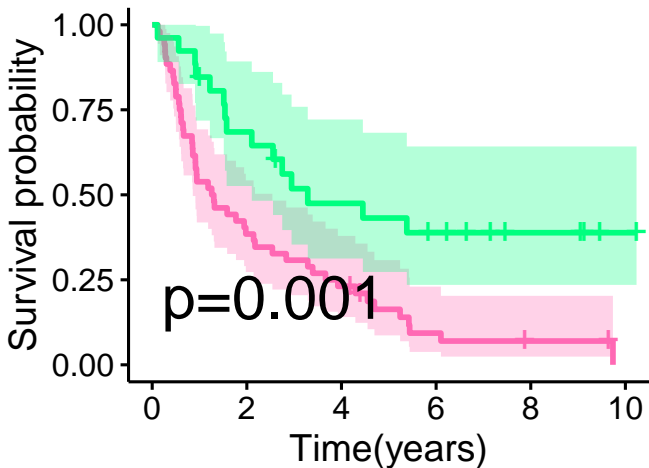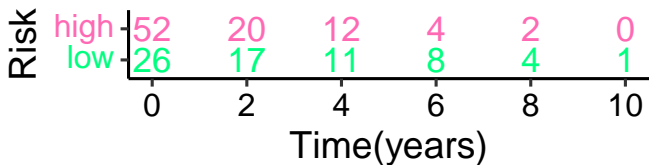

Supplement: Code S2 [file peerj-10-14506-s002.zip › 7. risk vs km/sur.M_M1.pdf]

# Patients with N0

Risk 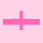 high 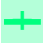 low

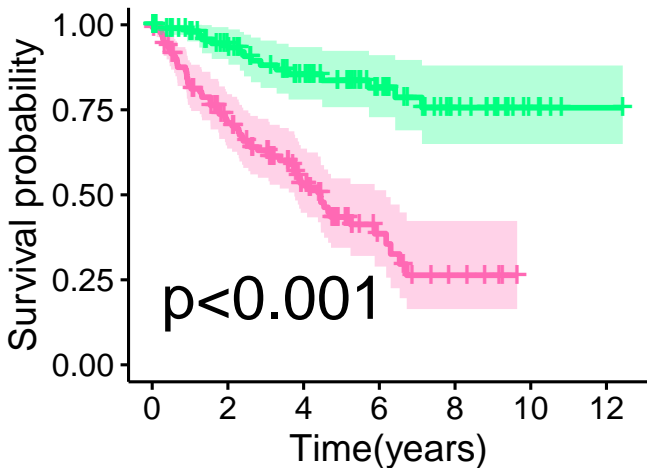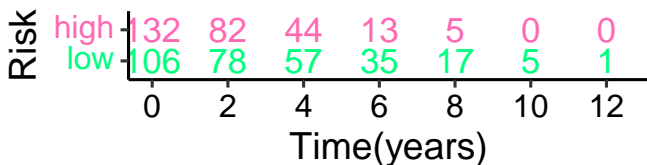

Supplement: Code S2 [file peerj-10-14506-s002.zip › 7. risk vs km/sur.N_N0.pdf]

# Patients with N1

Risk 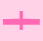 high 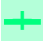 low

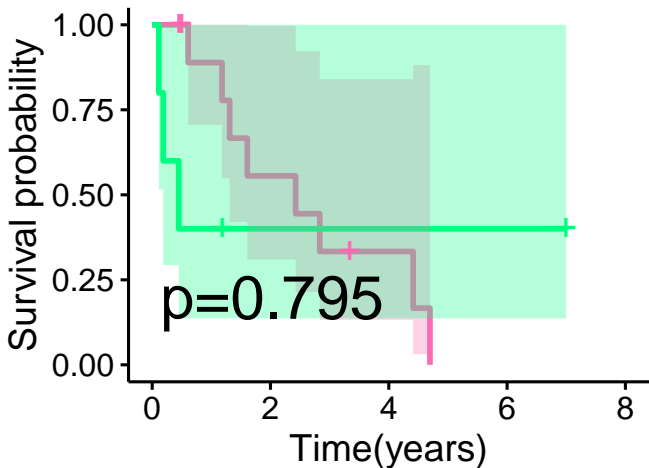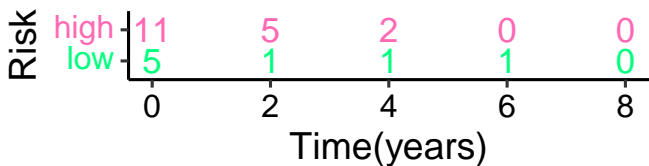

Supplement: Code S2 [file peerj-10-14506-s002.zip › 7. risk vs km/sur.N_N1.pdf]

# Patients with T1-2

Risk 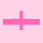 high 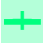 low

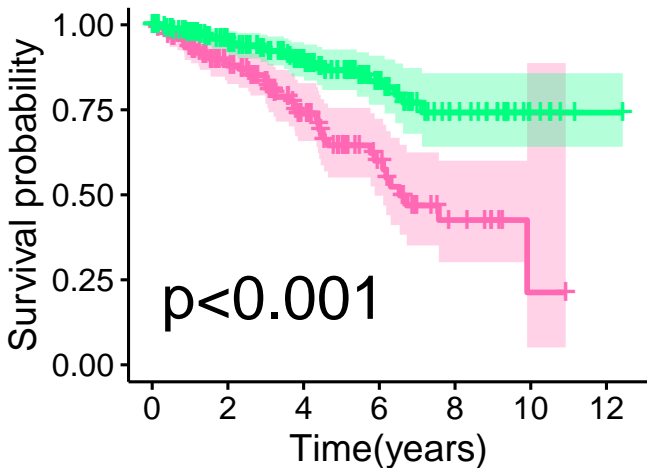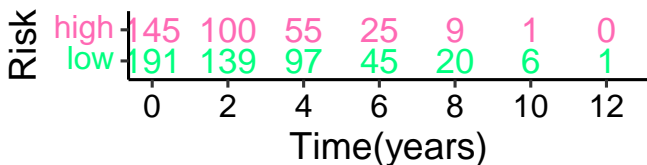

Supplement: Code S2 [file peerj-10-14506-s002.zip › 7. risk vs km/sur.T_T1-2.pdf]

# Patients with T3-4

Risk 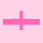 high 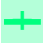 low

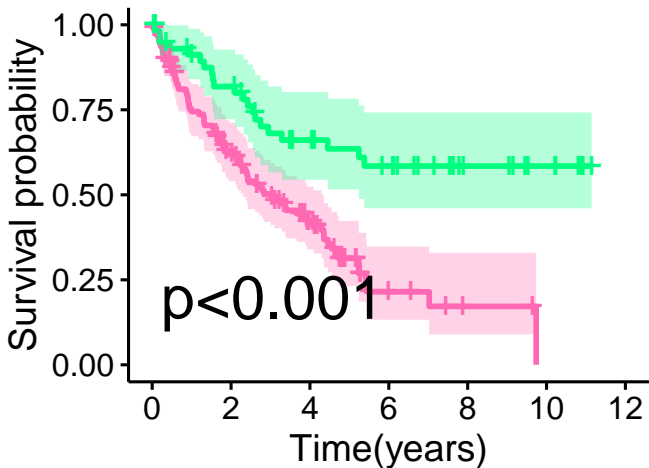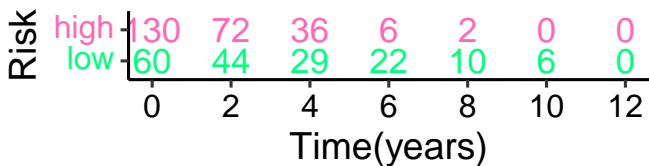

Supplement: Code S2 [file peerj-10-14506-s002.zip › 7. risk vs km/sur.T_T3-4.pdf]

# Patients with age>60 years

Risk 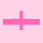 high 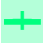 low

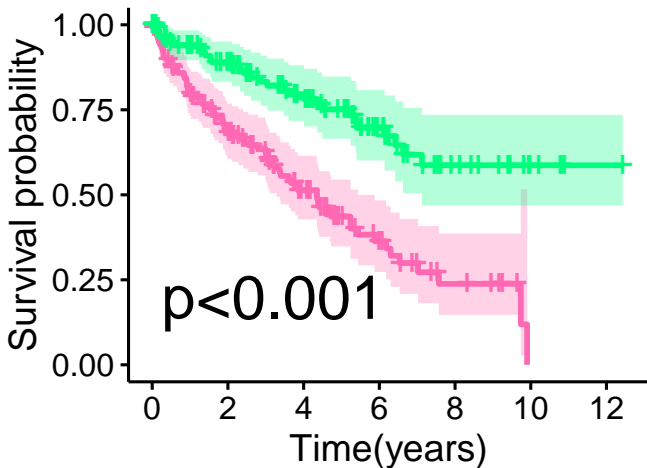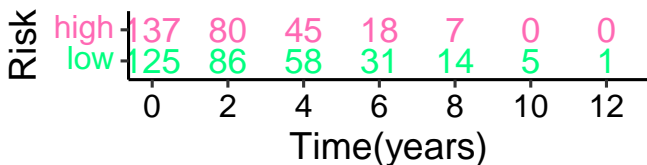

Supplement: Code S2 [file peerj-10-14506-s002.zip › 7. risk vs km/sur.age_gt60 years.pdf]

# Patients with age $\leq$ 60 years

Risk 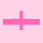 high 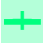 low

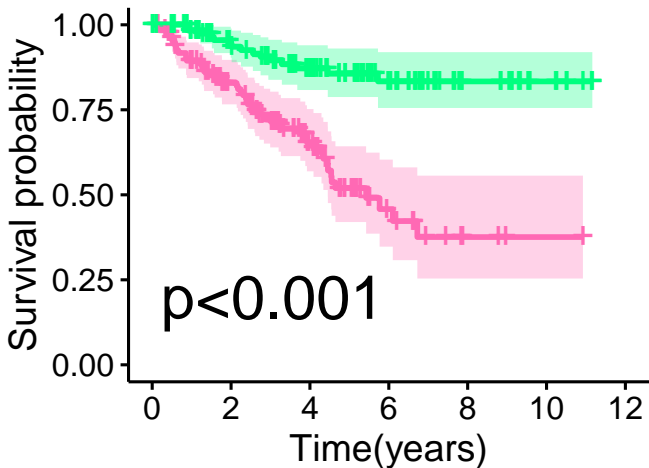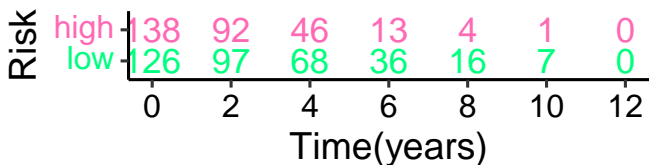

Supplement: Code S2 [file peerj-10-14506-s002.zip › 7. risk vs km/sur.age_le60 years.pdf]

# Patients with Female

Risk 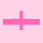 high 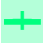 low

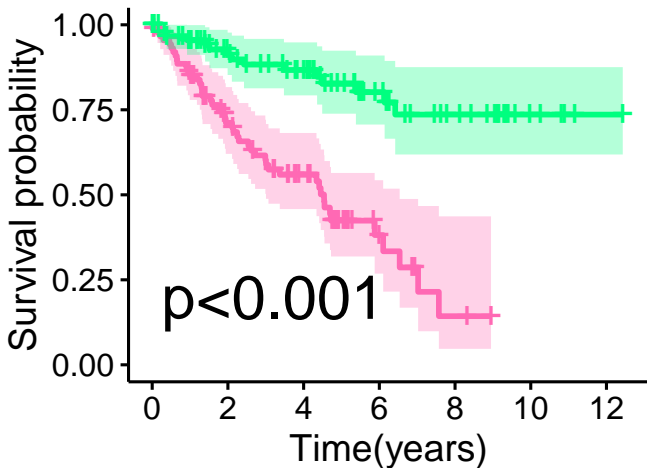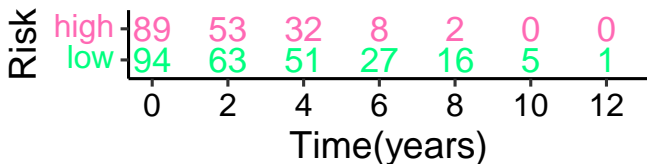

Supplement: Code S2 [file peerj-10-14506-s002.zip › 7. risk vs km/sur.gender_Female.pdf]

# Patients with Male

Risk 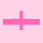 high 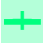 low

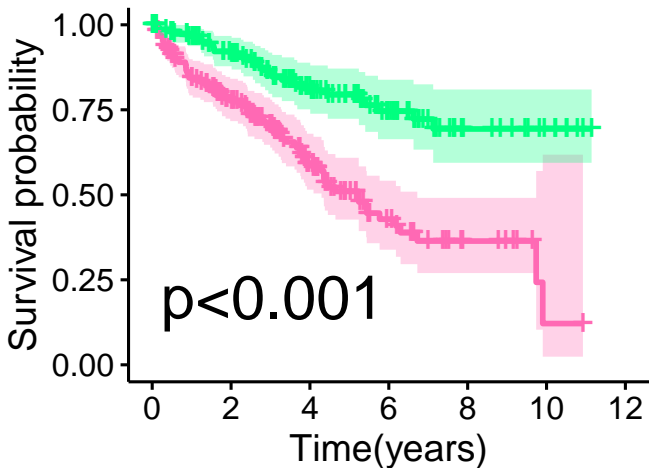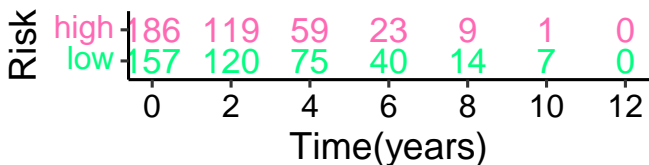

Supplement: Code S2 [file peerj-10-14506-s002.zip › 7. risk vs km/sur.gender_Male.pdf]

# Patients with G1-2

Risk 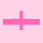 high 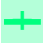 low

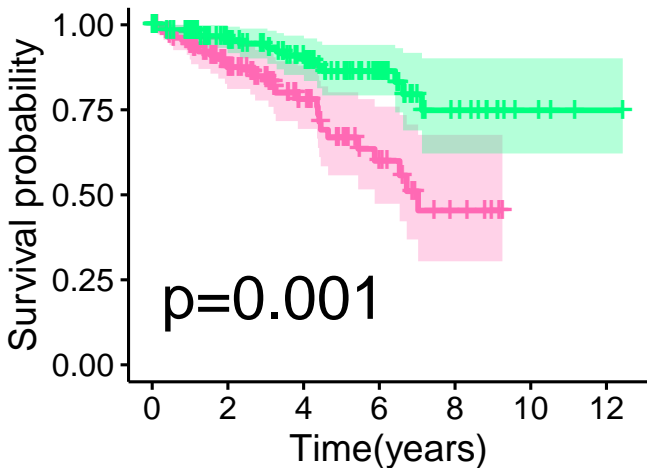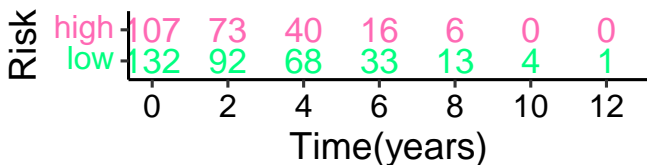

Supplement: Code S2 [file peerj-10-14506-s002.zip › 7. risk vs km/sur.grade_G1-2.pdf]

# Patients with G3-4

Risk 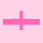 high 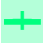 low

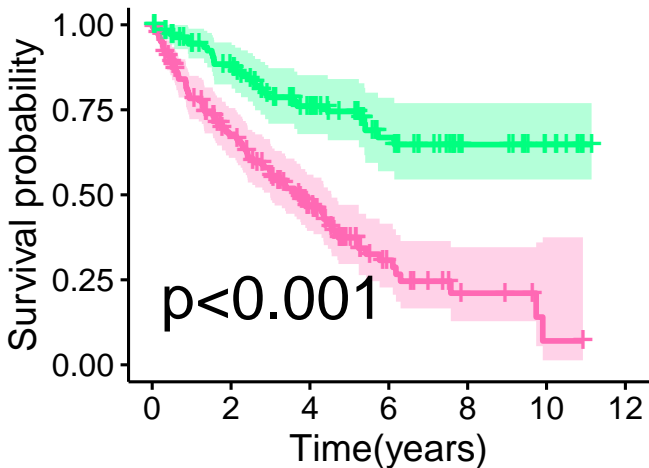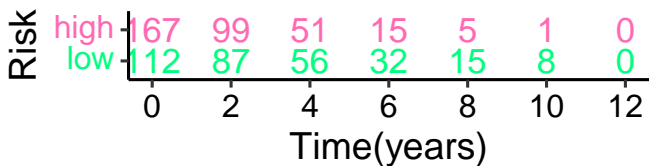

Supplement: Code S2 [file peerj-10-14506-s002.zip › 7. risk vs km/sur.grade_G3-4.pdf]

# Patients with Stage I–II

Risk 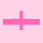 high 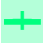 low

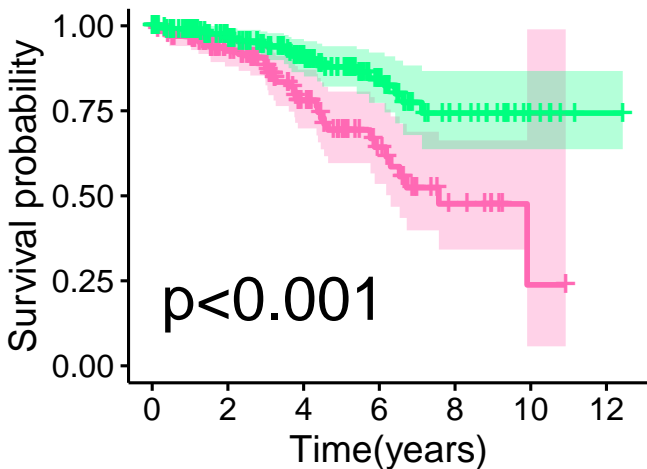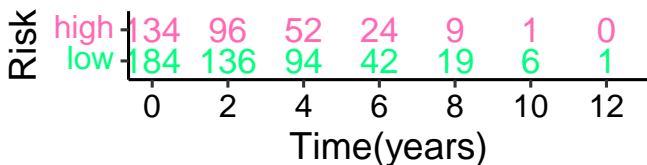

Supplement: Code S2 [file peerj-10-14506-s002.zip › 7. risk vs km/sur.stage_Stage I-II.pdf]

# Patients with Stage III–IV

Risk 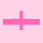 high 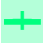 low

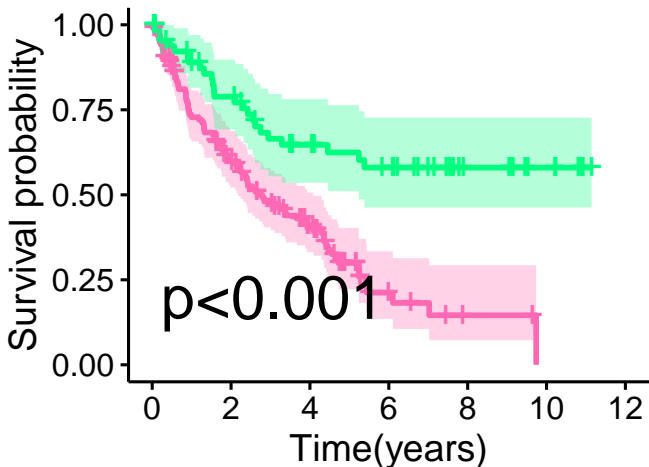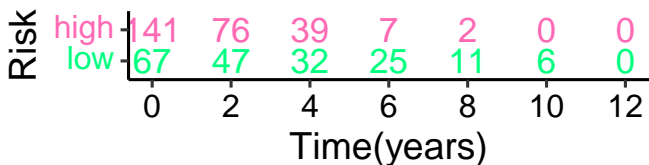

Supplement: Code S2 [file peerj-10-14506-s002.zip › 7. risk vs km/sur.stage_Stage III-IV.pdf]

Immune subtype C1 C2 C3 C4 C6

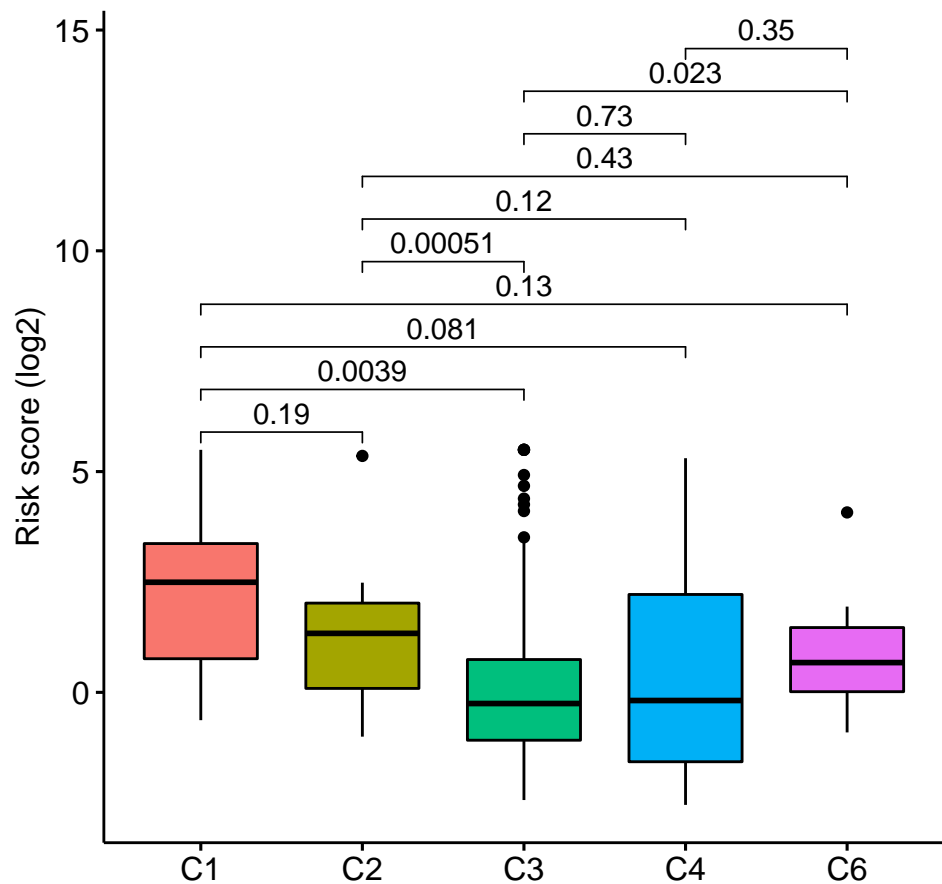

Supplement: Code S2 [file peerj-10-14506-s002.zip › 8. immuneSubytp/immuneSubtype.pdf]

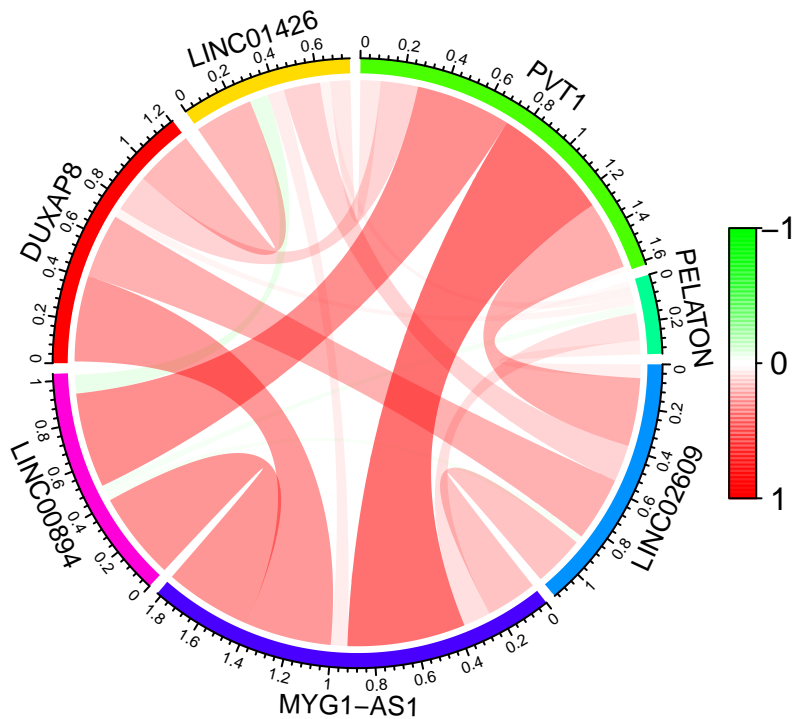

Supplement: Code S2 [file peerj-10-14506-s002.zip › 9. circus/risk.circos.pdf]

**A**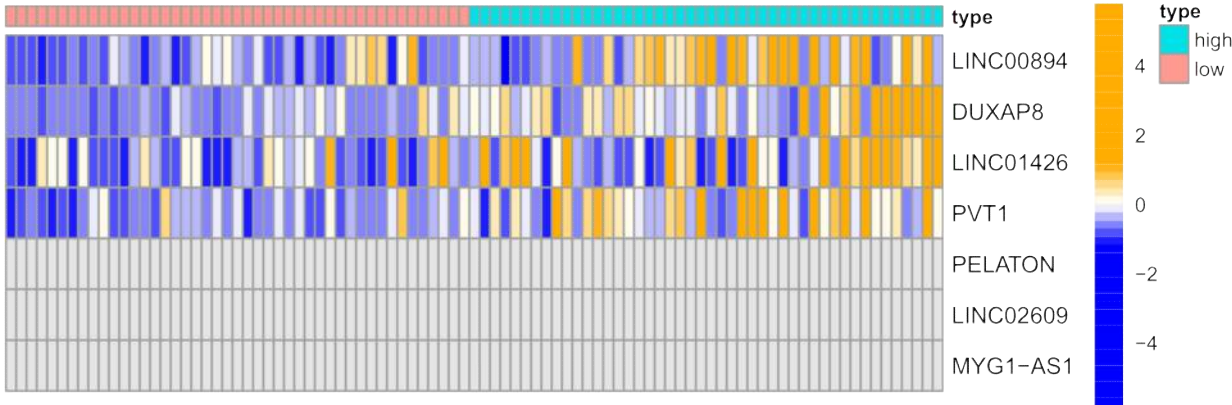**B**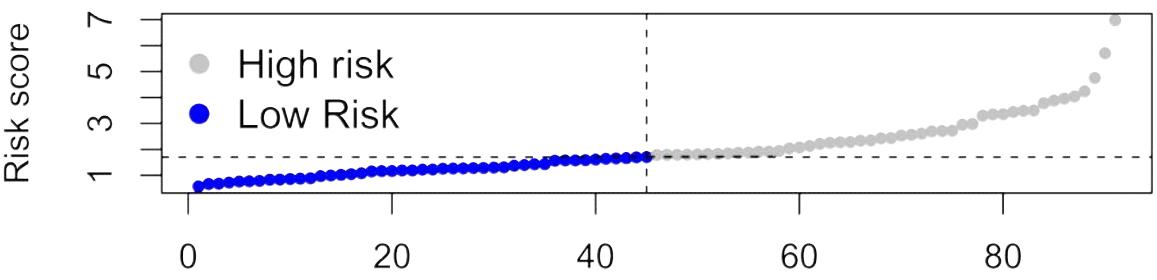**C**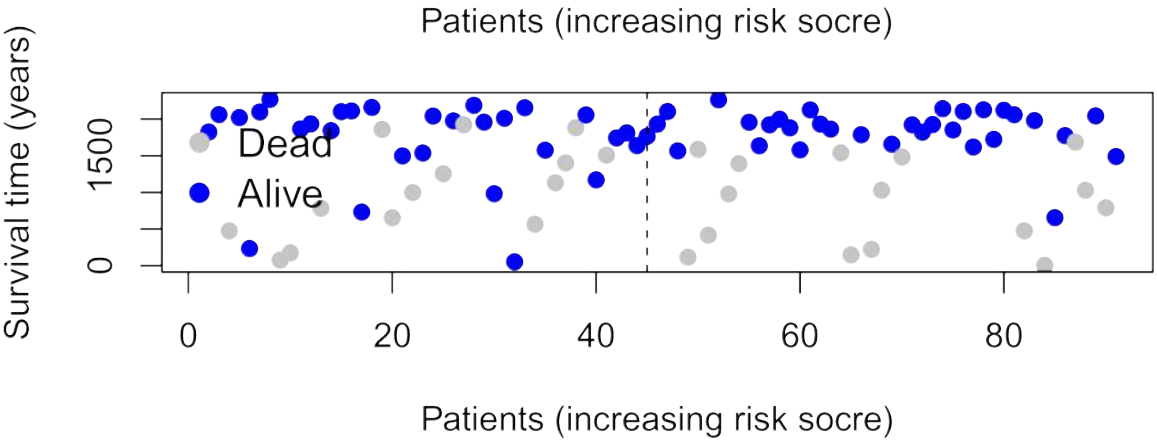**D**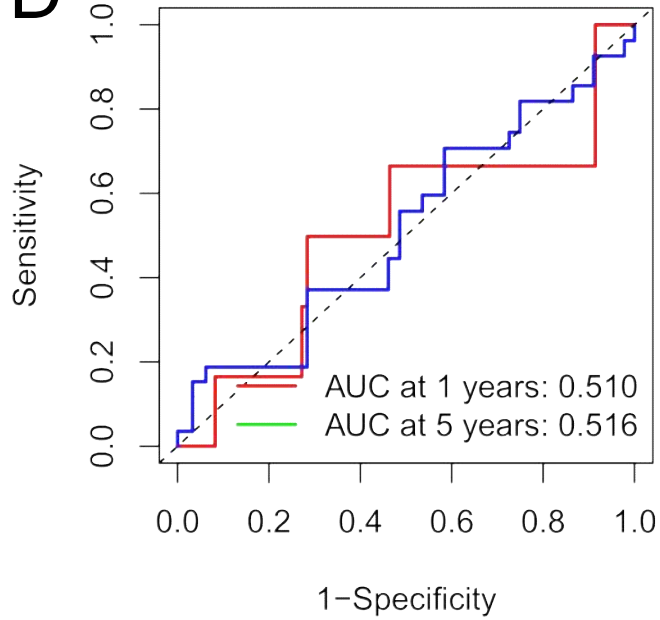

Supplement: Figure S2 — (A) Heatmap of FRlncRNA expression profiles showed the expression of FRlncRNAs in high-risk and low-risk groups in the ICGC database; (B) Risk score distribution plot showed the distribution of high-risk and low-risk in the ICGC database; (C) Scatter plot showed the correlation between the survival status and risk score in the ICGC database; (D) ROC curves and their AUC values showed 1-,and 5-year predictions in the ICGC database. [file peerj-10-14506-s007.pdf]
