# Supplementary material for: Identification and validation of a ferroptosis-related lncRNA signature to robustly predict the prognosis, immune microenvironment, and immunotherapy efficiency in patients with clear cell renal cell carcinoma
Source: PeerJ. 2022 Dec 19;10:e14506. doi: 10.7717/peerj.14506 (PMC9774008; doi:10.7717/peerj.14506)
Supplement: Table S1 [file peerj-10-14506-s004.docx]

**Supplementary Table 1. The comparison of ferroptosis-related lncRNA prognostic signatures studies for patients with ccRCC**

| Studies | Signature | Database | Number | Gene list | 5-year AUC value of OS |
| --- | --- | --- | --- | --- | --- |
| Xing et al (2021)[1] | Ferroptosis-related lncRNA | TCGA | 3 | DUXAP8, LINC02609, LUCAT1 | 0.7158 |
| Chen et al (2021)[2] | Ferroptosis-related lncRNA | TCGA  GEO | 7 | LINCO0460, LINC00941, LINC02027, AC027271.1, AC026401.3, AC124854.1 and AC020907.4 | 0.756 |
| Zhou, et al (2022)[3] | Ferroptosis-related lncRNA | TCGA  ICGC | 8 | AL590094.1, LINC00460, LINC00944, AC024060.1, HOXB-AS4, LINC01615, EPB41L4A-DT, LINC01550 | 0.764 |
| Bai et al (2022)[4] | Ferroptosis-related lncRNA | TCGA | 9 | AC026401.3, LINC01615, PRKAR1B-AS1, LINC02609, LINC00460, AC084876.1, AC008870.2, LINC02747, and AC103706.1 | none |
| Han et al (2022)[5] | Ferroptosis-and immune-related lncRNA | TCGA  ICGC | 3 | AC124854.1, LINC02609, and ZNF503-AS2 | 0.72 |
| Our study | Ferroptosis-related lncRNA | TCGA  ICGC  GEO | 7 | LINC00894, DUXAP8, LINC01426, PVT1, PELATON, LINC02609, MYG1-AS1 | 0.772 |

LncRNA, long non-coding RNA; TCGA, The Cancer Genome Atlas; AUC, area under the curve; OS, overall survival; ccRCC, clear cell renal cell carcinoma. ICGC, International Cancer Genome Consortium; GEO, Gene Expression Omnibus.

1. Xing XL, Yao ZY, Ou J, Xing C, Li F: Development and validation of ferroptosis-related lncRNAs prognosis signatures in kidney renal clear cell carcinoma. *Cancer Cell Int* 2021, 21(1):591.

2. Chen X, Tu J, Ma L, Huang Y, Yang C, Yuan X: Analysis of Ferroptosis-Related LncRNAs Signatures Associated with Tumor Immune Infiltration and Experimental Validation in Clear Cell Renal Cell Carcinoma. *Int J Gen Med* 2022, 15:3215-3235.

3. Zhou Z, Yang Z, Cui Y, Lu S, Huang Y, Che X, Yang L, Zhang Y: Identification and Validation of a Ferroptosis-Related Long Non-Coding RNA (FRlncRNA) Signature to Predict Survival Outcomes and the Immune Microenvironment in Patients With Clear Cell Renal Cell Carcinoma. *Front Genet* 2022, 13:787884.

4. Bai Z, Zhao Y, Yang X, Wang L, Yin X, Chen Y, Lu J: A Novel Prognostic Ferroptosis-Related Long Noncoding RNA Signature in Clear Cell Renal Cell Carcinoma. *J Oncol* 2022, 2022:6304824.

5. Han Z, Wang H, Liu Y, Xing XL: Establishment of a prognostic ferroptosis- and immune-related long noncoding RNAs profile in kidney renal clear cell carcinoma. *Front Genet* 2022, 13:915372.
