# Supplementary material for: Identification and validation of a ferroptosis-related lncRNA signature to robustly predict the prognosis, immune microenvironment, and immunotherapy efficiency in patients with clear cell renal cell carcinoma
Source: PeerJ. 2022 Dec 19;10:e14506. doi: 10.7717/peerj.14506 (PMC9774008; doi:10.7717/peerj.14506)
Supplement: Figure S1 [file peerj-10-14506-s006.pdf]

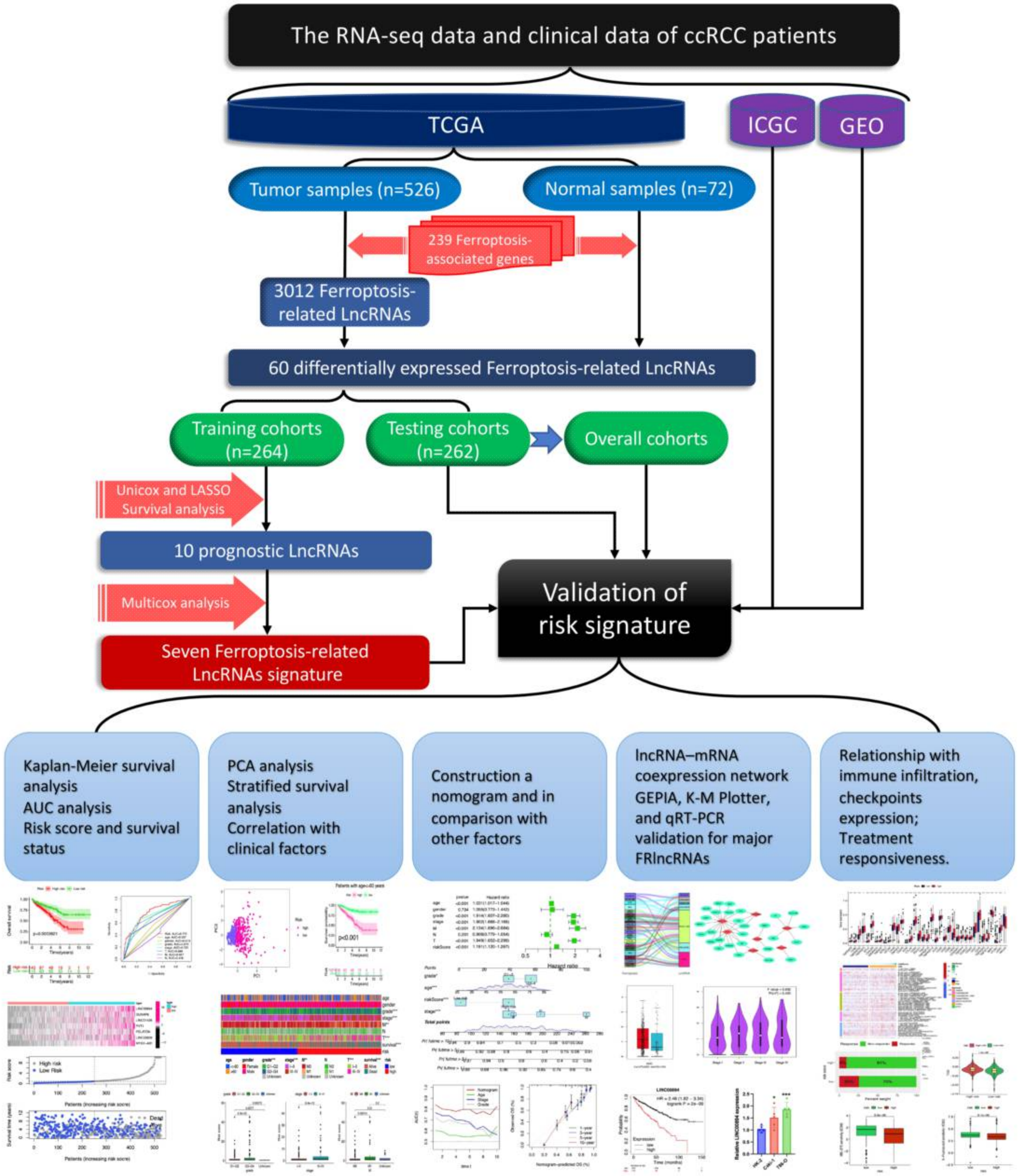
The RNA-seq data and clinical data of ccRCC patients
TCGA
ICGC
GEO
Tumor samples (n=526)
Normal samples (n=72)
239 Ferroptosis-associated genes
3012 Ferroptosis-related LncRNAs
60 differentially expressed Ferroptosis-related LncRNAs
Training cohorts (n=264)
Testing cohorts (n=262)
Overall cohorts
Unicox and LASSO Survival analysis
10 prognostic LncRNAs
Multicox analysis
Seven Ferroptosis-related LncRNAs signature
Validation of risk signature
Kaplan-Meier survival analysis AUC analysis Risk score and survival status
PCA analysis Stratified survival analysis Correlation with clinical factors
Construction a nomogram and in comparison with other factors
lncRNA–mRNA coexpression network GEPIA, K-M Plotter, and qRT-PCR validation for major FRlncRNAs
Relationship with immune infiltration, checkpoints expression; Treatment responsiveness.
