# Supplementary material for: Identification and validation of a ferroptosis-related lncRNA signature to robustly predict the prognosis, immune microenvironment, and immunotherapy efficiency in patients with clear cell renal cell carcinoma
Source: PeerJ. 2022 Dec 19;10:e14506. doi: 10.7717/peerj.14506 (PMC9774008; doi:10.7717/peerj.14506)
Supplement: Figure S3 — Univariate (A) and multivariate (B) Cox survival analysis showed that the risk signature was an independent prognostic factor, in training cohorts; Univariate (C) and multivariate (D) Cox survival analysis showed that the risk signature was an independent prognostic factor, in testing cohorts. [file peerj-10-14506-s008.pdf]

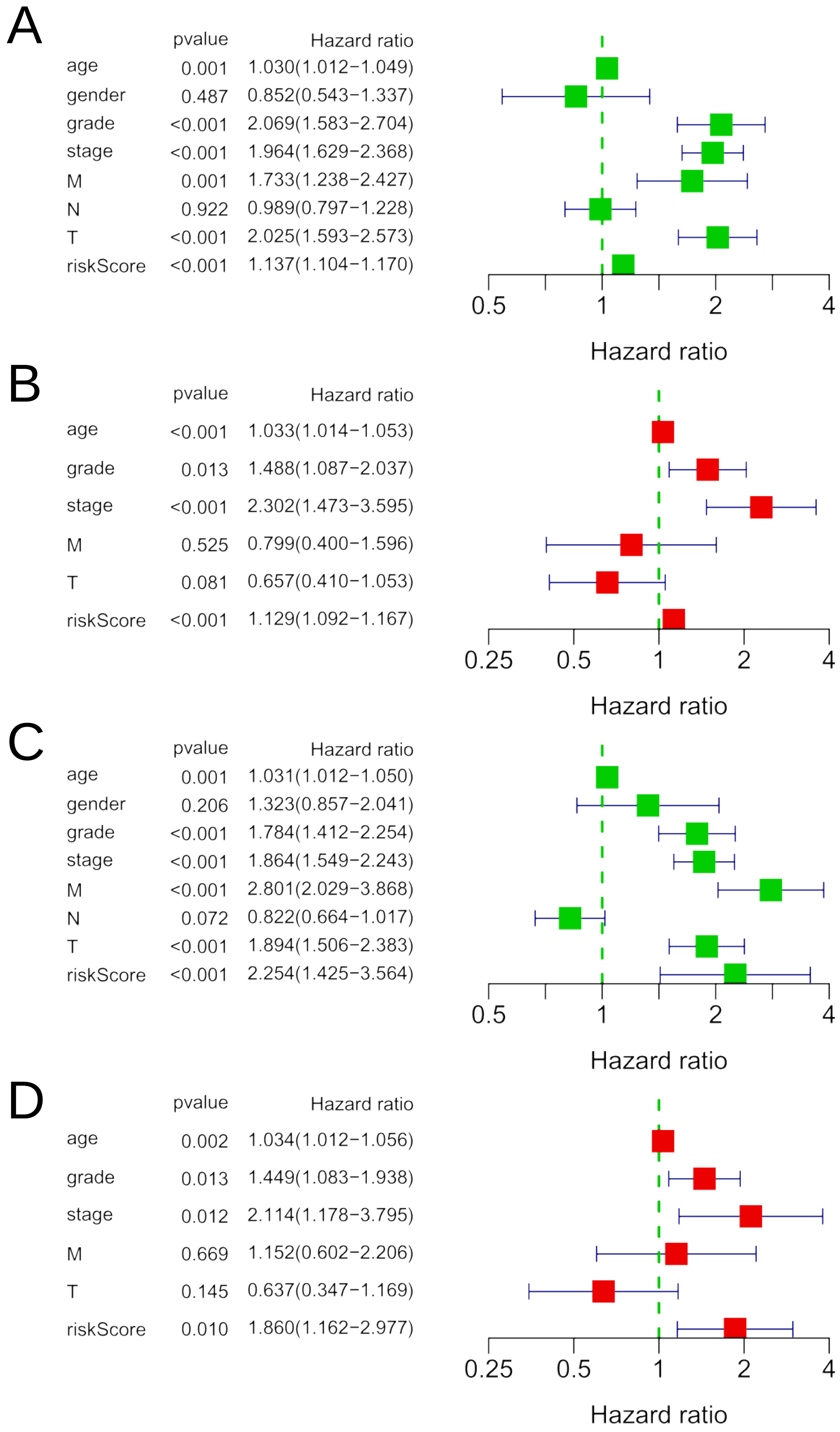
A
pvalue Hazard ratio
age 0.001 1.030(1.012−1.049)
gender 0.487 0.852(0.543−1.337)
grade <0.001 2.069(1.583−2.704)
stage <0.001 1.964(1.629−2.368)
M 0.001 1.733(1.238−2.427)
N 0.922 0.989(0.797−1.228)
T <0.001 2.025(1.593−2.573)
riskScore <0.001 1.137(1.104−1.170)
0.5 1 2 4
Hazard ratio
B
pvalue Hazard ratio
age <0.001 1.033(1.014−1.053)
grade 0.013 1.488(1.087−2.037)
stage <0.001 2.302(1.473−3.595)
M 0.525 0.799(0.400−1.596)
T 0.081 0.657(0.410−1.053)
riskScore <0.001 1.129(1.092−1.167)
0.25 0.5 1 2 4
Hazard ratio
C
pvalue Hazard ratio
age 0.001 1.031(1.012−1.050)
gender 0.206 1.323(0.857−2.041)
grade <0.001 1.784(1.412−2.254)
stage <0.001 1.864(1.549−2.243)
M <0.001 2.801(2.029−3.868)
N 0.072 0.822(0.664−1.017)
T <0.001 1.894(1.506−2.383)
riskScore <0.001 2.254(1.425−3.564)
0.5 1 2 4
Hazard ratio
D
pvalue Hazard ratio
age 0.002 1.034(1.012−1.056)
grade 0.013 1.449(1.083−1.938)
stage 0.012 2.114(1.178−3.795)
M 0.669 1.152(0.602−2.206)
T 0.145 0.637(0.347−1.169)
riskScore 0.010 1.860(1.162−2.977)
0.25 0.5 1 2 4
Hazard ratio
